# Supplementary figures and images for: Physical measures of physical functioning as prognostic factors to predict outcomes in low back pain: A systematic review and narrative synthesis
Source: PLoS One. 2025 Oct 28;20(10):e0335535. doi: 10.1371/journal.pone.0335535 (PMC12561921; doi:10.1371/journal.pone.0335535)

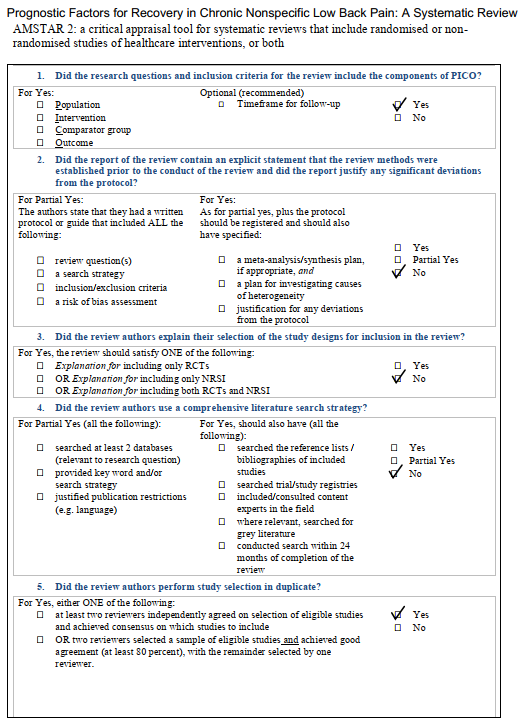


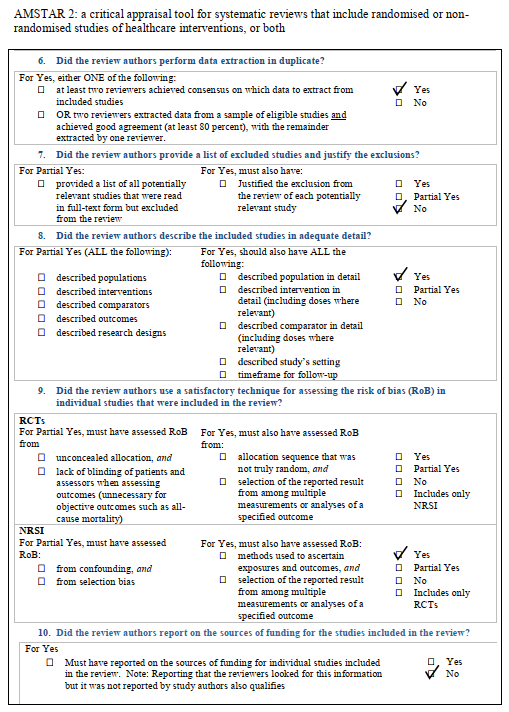


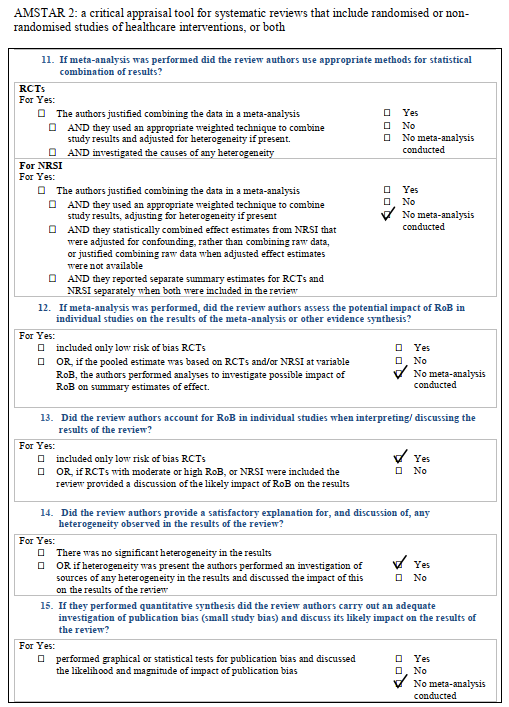


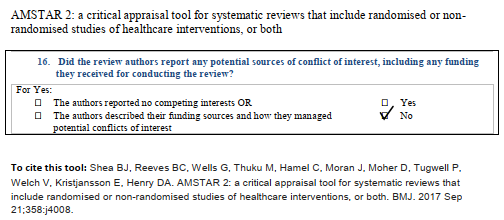


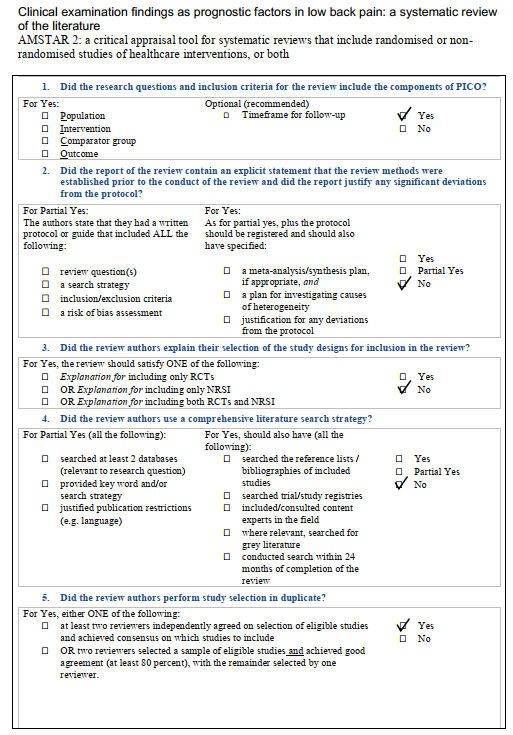


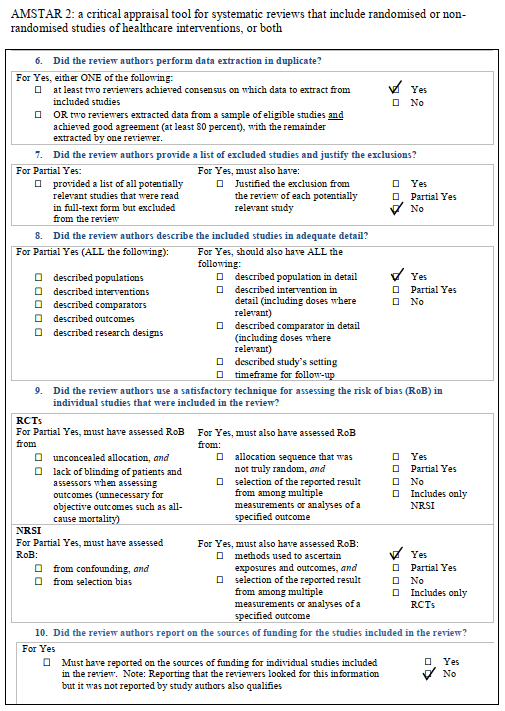


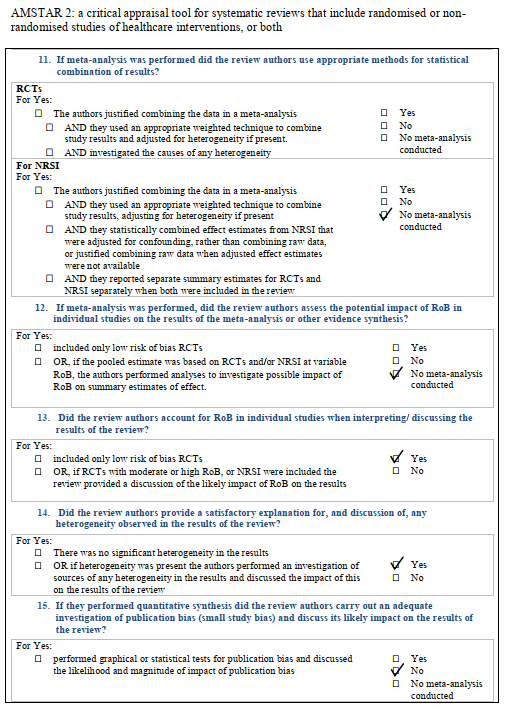


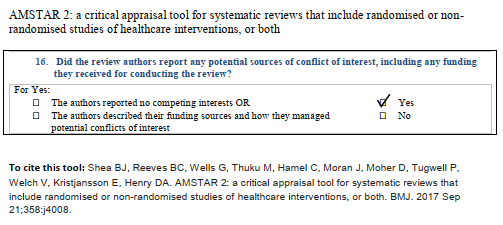

Supplement: S1 File — (DOCX) [file pone.0335535.s001.docx]
