# Supplementary material for: Physical measures of physical functioning as prognostic factors to predict outcomes in low back pain: A systematic review and narrative synthesis
Source: PLoS One. 2025 Oct 28;20(10):e0335535. doi: 10.1371/journal.pone.0335535 (PMC12561921; doi:10.1371/journal.pone.0335535)
Supplement: S2 File — (DOCX) [file pone.0335535.s002.docx]

**Ovid MEDLINE(R) ALL** 1946 to May 29, 2024 **N =** 7104

Search Strategy:

| **#** | **Searches** |
| --- | --- |
| 1 | Low Back Pain/ |
| 2 | (low* adj5 back adj5 (pain* or ache*)).tw,kf. |
| 3 | (backache* adj4 low*).tw,kf. |
| 4 | (backpain* adj4 low*).tw,kf. |
| 5 | (lumbar or lumbosacral or lumbo-sacral or low* back*).tw,kf. |
| 6 | (lumbago or sciatica).tw,kf. |
| 7 | (radiculopathy or radiculitis or radicular pain*).tw,kf. |
| 8 | 1 or 2 or 3 or 4 or 5 or 6 or 7 |
| 9 | (physical outcome measur* or physical measures of function*or measures of function* or physical function* test).tw,kf. |
| 10 | (range of motion or ROM).tw,kf. |
| 11 | Schober.tw,kf. |
| 12 | (finger* adj3 (floor or toe or knee or fibular head)).tw,kf. |
| 13 | (wrist crease adj3 floor).tw,kf. |
| 14 | (straight leg raise* or SLR or Lasegue*).tw,kf. |
| 15 | (isometric strength or isokinetic strength).tw,kf. |
| 16 | (motor control or movement control or functional movement screen* or FMS or sitting one leg knee extension or posterior pelvic tilt or waiter's bow or one leg stance).tw,kf. |
| 17 | gait/ or gait analysis/ |
| 18 | walking speed/ |
| 19 | (spatiotemporal gait or spatio temporal gait or stride length or stride duration or gait speed or cadence or gait asymmetry or stance phase or swing phase or double limb support or single limb support).tw,kf. |
| 20 | (inclinometer or goniometer or kyphometer or electromagnetic tracking).tw,kf. |
| 21 | muscle strength dynamometer/ |
| 22 | (dynamometer or manual muscle test or MedX or Cybex or Kin-Com or RehaGait or JAMAR).tw,kf. |
| 23 | (aerobic capacity or VO2* or bicycle ergomet* or maximal graded exercise* or steep ramp).tw,kf. |

| 24 | (Biering Sorensen or modified Sorensen).tw,kf. |
| --- | --- |
| 25 | Roman chair.tw,kf. |
| 26 | (sternum adj3 (ground or floor)).tw,kf. |
| 27 | muscle endurance.tw,kf. |
| 28 | ((back or body or trunk) adj3 endurance).tw,kf. |
| 29 | prone bridge.tw,kf. |
| 30 | ((lower extremities or (hips and knees)) adj5 (90deg* or "90 degree*")).tw,kf. |
| 31 | (arch-up* or sit-up* or squat* or dumbbell press*).tw,kf. |
| 32 | (double limb stance or single limb stance or stork stand* or flamingo balance or y-balance).tw,kf. |
| 33 | (clinical test* adj2 sensory interaction adj2 balance).tw,kf. |
| 34 | (Berg balance scale or Tinetti* or performance oriented mobility assessment* or tandem walk*).tw,kf. |
| 35 | lower extremity motor coordination test*.tw,kf. |
| 36 | (chair adj3 (stand* or rise*)).tw,kf. |
| 37 | (sit to stand or stand up or stand ups or roll*).tw,kf. |
| 38 | (lie adj2 sit).tw,kf. |
| 39 | (bed adj2 chair).tw,kf. |
| 40 | step*.tw,kf. |
| 41 | lifting/ |
| 42 | (lift* or progressive isoinertial lifting evaluation or pile).tw,kf. |
| 43 | (forward reach or functional reach).tw,kf. |
| 44 | walk test/ |
| 45 | (self-paced walk* or 4-meter walk* or 4-metre walk* or 5-meter walk* or 5-metre walk* or 10-meter walk* or 10-metre walk* or 15-meter walk* or 15-metre walk* or 50-meter walk* or 50-metre walk* or 50-foot walk* or 5-minute walk* or 6- minute walk* or treadmill or overground walk*).tw,kf. |
| 46 | (walk adj3 hall*).tw,kf. |
| 47 | shuttle walk*.tw,kf. |
| 48 | (stair* adj2 climb*).tw,kf. |
| 49 | ("timed up and go" or TUG or "8 foot up and go").tw,kf. |
| 50 | accelerometry/ |
| 51 | wearable electronic devices/ or fitness trackers/ |
| 52 | (acceleromet* or activity monitor or pedomet* or GPS or watch or smartwatch).tw,kf. |
| 53 | (6WT adj3 app*).tw,kf. |
| 54 | functional capacity evaluation.tw,kf. |

| 55 | 9 or 10 or 11 or 12 or 13 or 14 or 15 or 16 or 17 or 18 or 19 or 20 or 21 or 22 or 23 or 24 or 25 or 26  or 27 or 28 or 29 or 30 or 31 or 32 or 33 or 34 or 35 or 36 or 37 or 38 or 39 or 40 or 41 or 42 or 43 or  44 or 45 or 46 or 47 or 48 or 49 or 50 or 51 or 52 or 53 or 54 |
| --- | --- |
| 56 | exp Risk/ |
| 57 | risk.tw. |
| 58 | exp Cohort Studies/ |
| 59 | cohort.tw. |
| 60 | exp Prognosis/ |
| 61 | "prognos*".tw. |
| 62 | "predict*".tw. |
| 63 | exp Incidence/ |
| 64 | incidence.tw. |
| 65 | exp Survival Analysis/ |
| 66 | survival.tw. |
| 67 | "causal factor".tw. |
| 68 | course.tw. |
| 69 | 56 or 57 or 58 or 59 or 60 or 61 or 62 or 63 or 64 or 65 or 66 or 67 or 68 |
| 70 | 8 and 55 and 69 |

**Embase Classic+Embase**1947 to May 29, 2024 **N**= 2345
Search Strategy:

| **#** | **Searches** |
| --- | --- |
| 1 | follow up.mp. |
| 2 | prognos:.tw. |
| 3 | ep.fs. |
| 4 | Low Back Pain/ |
| 5 | (low* adj5 back adj5 (pain* or ache*)).tw,kf. |
| 6 | (backache* adj4 low*).tw,kf. |
| 7 | (backpain* adj4 low*).tw,kf. |
| 8 | 4 or 5 or 6 or 7 |
| 9 | (range of motion or ROM).tw,kf. |
| 10 | Schober.tw,kf. |
| 11 | (finger* adj3 (floor or toe or knee or fibular head)).tw,kf. |
| 12 | (wrist crease adj3 floor).tw,kf. |
| 13 | (straight leg raise* or SLR or Lasegue*).tw,kf. |
| 14 | (isometric strength or isokinetic strength).tw,kf. |
| 15 | (motor control or movement control or functional movement screen* or FMS or sitting one leg knee extension or posterior pelvic tilt or waiter's bow or one leg stance).tw,kf. |
| 16 | gait/ or gait analysis/ |
| 17 | walking speed/ |
| 18 | (spatiotemporal gait or spatio temporal gait or stride length or stride duration or gait speed or cadence or gait asymmetry or stance phase or swing phase or double limb support or single limb support).tw,kf. |
| 19 | (inclinometer or goniometer or kyphometer or electromagnetic tracking).tw,kf. |
| 20 | muscle strength dynamometer/ |
| 21 | (dynamometer or manual muscle test or MedX or Cybex or Kin-Com or RehaGait or JAMAR).tw,kf. |
| 22 | (aerobic capacity or VO2* or bicycle ergomet* or maximal graded exercise* or steep ramp).tw,kf. |
| 23 | (Biering Sorensen or modified Sorensen).tw,kf. |
| 24 | Roman chair.tw,kf. |
| 25 | (sternum adj3 (ground or floor)).tw,kf. |
| 26 | muscle endurance.tw,kf. |
| 27 | ((back or body or trunk) adj3 endurance).tw,kf. |
| 28 | prone bridge.tw,kf. |
| 29 | ((lower extremities or (hips and knees)) adj5 (90deg* or "90 degree*")).tw,kf. |
| 30 | (arch-up* or sit-up* or squat* or dumbbell press*).tw,kf. |
| 31 | (double limb stance or single limb stance or stork stand* or flamingo balance or y-balance).tw,kf. |
| 32 | (clinical test* adj2 sensory interaction adj2 balance).tw,kf. |
| 33 | (Berg balance scale or Tinetti* or performance oriented mobility assessment* or tandem walk*).tw,kf. |
| 34 | lower extremity motor coordination test*.tw,kf. |
| 35 | (chair adj3 (stand* or rise*)).tw,kf. |
| 36 | (sit to stand or stand up or stand ups or roll*).tw,kf. |
| 37 | (lie adj2 sit).tw,kf. |
| 38 | (bed adj2 chair).tw,kf. |
| 39 | step*.tw,kf. |
| 40 | functional capacity evaluation.tw,kf. |
| 41 | lifting/ |
| 42 | (lift* or progressive isoinertial lifting evaluation or pile).tw,kf. |
| 43 | (forward reach or functional reach).tw,kf. |
| 44 | walk test/ |
| 45 | (self-paced walk* or 4-meter walk* or 4-metre walk* or 5-meter walk* or 5-metre walk* or 10-meter walk* or 10-metre walk* or 15-meter walk* or 15-metre walk* or 50-meter walk* or 50-metre walk* or 50-foot walk* or 5-minute walk* or 6-minute walk* or treadmill or overground walk*).tw,kf. |
| 46 | (walk adj3 hall*).tw,kf. |
| 47 | shuttle walk*.tw,kf. |
| 48 | (stair* adj2 climb*).tw,kf. |
| 49 | ("timed up and go" or TUG or "8 foot up and go").tw,kf. |
| 50 | accelerometry/ |
| 51 | wearable electronic devices/ or fitness trackers/ |
| 52 | (acceleromet* or activity monitor or pedomet* or GPS or watch or smartwatch).tw,kf. |
| 53 | (6WT adj3 app*).tw,kf. |
| 54 | 9 or 10 or 11 or 12 or 13 or 14 or 15 or 16 or 17 or 18 or 19 or 20 or 21 or 22 or 23 or 24 or 25 or 26 or 27 or 28 or 29 or 30 or 31 or 32 or 33 or 34 or 35 or 36 or 37 or 38 or 39 or 40 or 41 or 42 or 43 or 44 or 45 or 46 or 47 or 48 or 49 or 50 or 51 or 52 or 53 |
| 55 | exp outcome assessment, health care/ |
| 56 | outcome assessment.ti,ab,kf. |
| 57 | exp health status indicators/ |
| 58 | (physical function* or physical activit* or activity limitation*).tw,kf. |
| 59 | (physical outcome measures* or physical measures of function or physical measures or physical function test* or physical assessment).tw,kf. |
| 60 | (prognostic factors* or predictive factors* or baseline factors).tw,kf. |
| 61 | (outcomes* or results* or endpoints* or future findings*).tw,kf. |
| 62 | 55 or 56 or 57 or 58 or 59 or 60 or 61 |
| 63 | 1 or 2 or 3 |
| 64 | 8 and 54 and 63 |

**CINAHL (EBSCOhost) run** May 29, 2024 **– N=** 2281

| **#** | **Query** |
| --- | --- |
| S272 | S227 AND S270 AND S271 |
| S271 | S236 OR S269 |
| S270 | TI prognos* or AB prognos* |
| S269 | S237 OR S238 OR S239 OR S240 OR S241 OR S242 OR S243 OR S244 OR S245 OR S246 OR S247 OR S248 OR S249 OR S250 OR S251 OR S252 OR S253 OR S254 OR S255 OR S256 OR S257 OR S258 OR S259 OR S260 OR S261 OR S262 OR S263 OR S264 OR S265 OR S266 OR S267 OR S268 |
| S268 | TI (Physiotherapy functional mobility profile OR Barthel index) OR AB (Physiotherapy functional mobility profile OR Barthel index) |
| S267 | (MH "Barthel Index") |
| S266 | TI Activity measure for post-acute care 6 clicks OR AB Activity measure for post-acute care 6 clicks |
| S265 | TI ("timed up and go" OR TUG OR "8 foot up and go" OR Physical capability assessment tool OR PCAT OR aggregated functional performance test OR aggregated assessment of physical function OR short physical performance battery OR cumulated ambulation score OR functional independence measure OR Katz ADL index) OR AB ("timed up and go" OR TUG OR "8 foot up and go" OR Physical capability assessment tool OR PCAT OR aggregated functional performance test OR aggregated assessment of physical function OR short physical performance battery OR cumulated ambulation score OR functional independence measure OR Katz ADL index) |
| S264 | TI (stair* N2 climb*) OR AB (stair* N2 climb*) |
| S263 | (MH "Stair Climbing") |
| S262 | TI shuttle walk* OR AB shuttle walk* |
| S261 | TI (walk N3 hall*) OR AB (walk N3 hall*) |
| S260 | TI (self-paced walk* OR 4-meter walk* OR 4-metre walk* OR 5-meter walk* OR 5-metre walk* OR 10-meter walk* OR 10-metre walk* OR 15-meter walk* OR 15-metre walk* OR 50-meter walk* OR 50-metre walk* OR 50-foot walk* OR 5-minute walk* OR 6-minute walk* OR treadmill OR overground walk*) OR AB (self-paced walk* OR 4-meter walk* OR 4-metre walk* OR 5-meter walk* OR 5-metre walk* OR 10-meter walk* OR 10-metre walk* OR 15-meter walk* OR 15-metre walk* OR 50-meter walk* OR 50-metre walk* OR 50-foot walk* OR 5-minute walk* OR 6-minute walk* OR treadmill OR overground walk*) |
| S259 | TI (lift* OR progressive isoinertial lifting evaluation OR pile OR forward reach OR functional reach) OR AB (lift* OR progressive isoinertial lifting evaluation OR pile OR forward reach OR functional reach) |
| S258 | (MH "Reaching") |
| S257 | (MH "Lifting") |
| S256 | TI functional capacity evaluation OR AB functional capacity evaluation |
| S255 | TI ((stand OR standing) N2 continuous) OR AB (stand OR standing) N2 continuous) |
| S254 | TI step* OR AB step* |
| S253 | (MH "Step") |
| S252 | TI (bed N2 chair) OR AB (bed N2 chair) |
| S251 | TI (lie N2 sit) OR AB (lie N2 sit) |
| S250 | TI (sit to stand OR stand up OR stand ups OR roll*) OR AB (sit to stand OR stand up OR stand ups OR roll*) |
| S249 | TI (chair N3 (stand* OR rise*)) OR AB (chair N3 (stand* OR rise*)) |
| S248 | TI (Berg balance scale OR Tinetti* OR performance oriented mobility assessment* OR tandem walk* OR lower extremity motor coordination test*) OR AB (Berg balance scale OR Tinetti* OR performance oriented mobility assessment* OR tandem walk* OR lower extremity motor coordination test*) |
| S247 | TI (chair N3 (stand* OR rise*)) OR AB (chair N3 (stand* OR rise*)) |
| S246 | TI (clinical test* N2 sensory interaction N2 balance) OR AB (clinical test* N2 sensory interaction N2 balance) |
| S245 | TI (arch-up* OR sit-up* OR squat* OR dumbbell press* OR double limb stance OR single limb stance OR stork stand* OR flamingo balance OR y-balance) OR AB (arch-up* OR sit-up* OR squat* OR dumbbell press* OR double limb stance OR single limb stance OR stork stand* OR flamingo balance OR y-balance) |
| S244 | TI CTSIB OR AB CTSIB |
| S243 | TI ((lower extremities OR (hips AND knees)) N5 (90deg* OR "90 degree*")) OR AB ((lower extremities OR (hips AND knees)) N5 (90deg* OR "90 degree*")) |
| S242 | (MH "One Leg Stand") |
| S241 | TI prone bridge OR AB prone bridge |
| S240 | TI ((back OR body OR trunk) N3 endurance) OR AB ((back OR body OR trunk) N3 endurance) |
| S239 | TI (sternum N3 (ground OR floor)) OR AB (sternum N3 (ground OR floor)) |
| S238 | TI (aerobic capacity OR VO2* OR bicycle ergomet* OR maximal graded exercise* OR steep ramp OR muscle endurance OR Biering Sorensen OR modified Sorensen OR Roman chair) OR AB (aerobic capacity OR VO2* OR bicycle ergomet* OR maximal graded exercise* OR steep ramp OR muscle endurance OR Biering Sorensen OR modified Sorensen OR Roman chair) |
| S237 | (MH "Aerobic Capacity") |
| S236 | S228 OR S229 OR S230 OR S231 OR S232 OR S233 OR S234 OR S235 |
| S235 | TI lying or AB lying |
| S234 | TI (time N5 stand*) OR AB (time N5 stand*) |
| S233 | (MH "Exercise Intensity") |
| S232 | (MH "Accelerometers") OR (MH "Accelerometry") |
| S231 | TI (constant postures OR active postures OR sedentary postures OR sedentary activity OR walking time OR walking distance OR claudication index OR walking speed OR daily walking events OR light intensity OR moderate intensity OR vigorous intensity OR activity count OR gait cycles OR gait posture index OR physical activity OR acceleromet*) OR AB (constant postures OR active postures OR sedentary postures OR sedentary activity OR walking time OR walking distance OR claudication index OR walking speed OR daily walking events OR light intensity OR moderate intensity OR vigorous intensity OR activity count OR gait cycles OR gait posture index OR physical activity OR acceleromet*) |
| S230 | (MH "Fitness Trackers") OR (MH "Pedometers") |
| S229 | TI (activity monitor OR pedomet* OR GPS OR watch OR smartwatch) OR AB (activity monitor OR pedomet* OR GPS OR watch OR smartwatch) |
| S228 | TI (6WT N3 app*) OR AB (6WT N3 app*) |
| S227 | S219 OR S220 OR S221 OR S222 OR S223 OR S224 OR S225 OR S226 |
| S226 | (back* OR lumbosacral OR lumbo-sacral OR lumbar) |
| S225 | (radiculopathy OR radiculitis OR radicular pain*) |
| S224 | (lumbago OR sciatica) |
| S223 | (lumbar OR lumbosacral OR lumbo-sacral OR low* back*) |
| S222 | (lumbar vertebra* OR lumbar spin* OR lumbar disk OR lumbar disc) |
| S221 | "(lumbar vertebra* OR lumbar spin* OR lumbar disk OR lumbar disc)" |
| S220 | (MH "Sciatica") |
| S219 | (MH "Lumbar Vertebrae") |
| S218 | S172 AND S215 AND S216 |
| S216 | S181 OR S214 |
| S215 | TI prognos* or AB prognos* |
| S214 | S182 OR S183 OR S184 OR S185 OR S186 OR S187 OR S188 OR S189 OR S190 OR S191 OR S192 OR S193 OR S194 OR S195 OR S196 OR S197 OR S198 OR S199 OR S200 OR S201 OR S202 OR S203 OR S204 OR S205 OR S206 OR S207 OR S208 OR S209 OR S210 OR S211 OR S212 OR S213 |
| S213 | TI (Physiotherapy functional mobility profile OR Barthel index) OR AB (Physiotherapy functional mobility profile OR Barthel index) |
| S212 | (MH "Barthel Index") |
| S211 | TI Activity measure for post-acute care 6 clicks OR AB Activity measure for post-acute care 6 clicks |
| S210 | TI ("timed up and go" OR TUG OR "8 foot up and go" OR Physical capability assessment tool OR PCAT OR aggregated functional performance test OR aggregated assessment of physical function OR short physical performance battery OR cumulated ambulation score OR functional independence measure OR Katz ADL index) OR AB ("timed up and go" OR TUG OR "8 foot up and go" OR Physical capability assessment tool OR PCAT OR aggregated functional performance test OR aggregated assessment of physical function OR short physical performance battery OR cumulated ambulation score OR functional independence measure OR Katz ADL index) |
| S209 | TI (stair* N2 climb*) OR AB (stair* N2 climb*) |
| S208 | (MH "Stair Climbing") |
| S207 | TI shuttle walk* OR AB shuttle walk* |
| S206 | TI (walk N3 hall*) OR AB (walk N3 hall*) |
| S205 | TI (self-paced walk* OR 4-meter walk* OR 4-metre walk* OR 5-meter walk* OR 5-metre walk* OR 10-meter walk* OR 10-metre walk* OR 15-meter walk* OR 15-metre walk* OR 50-meter walk* OR 50-metre walk* OR 50-foot walk* OR 5-minute walk* OR 6-minute walk* OR treadmill OR overground walk*) OR AB (self-paced walk* OR 4-meter walk* OR 4-metre walk* OR 5-meter walk* OR 5-metre walk* OR 10-meter walk* OR 10-metre walk* OR 15-meter walk* OR 15-metre walk* OR 50-meter walk* OR 50-metre walk* OR 50-foot walk* OR 5-minute walk* OR 6-minute walk* OR treadmill OR overground walk*) |
| S204 | TI (lift* OR progressive isoinertial lifting evaluation OR pile OR forward reach OR functional reach) OR AB (lift* OR progressive isoinertial lifting evaluation OR pile OR forward reach OR functional reach) |
| S203 | (MH "Reaching") |
| S202 | (MH "Lifting") |
| S201 | TI functional capacity evaluation OR AB functional capacity evaluation |
| S200 | TI ((stand OR standing) N2 continuous) OR AB (stand OR standing) N2 continuous) |
| S199 | TI step* OR AB step* |
| S198 | (MH "Step") |
| S197 | TI (bed N2 chair) OR AB (bed N2 chair) |
| S196 | TI (lie N2 sit) OR AB (lie N2 sit) |
| S195 | TI (sit to stand OR stand up OR stand ups OR roll*) OR AB (sit to stand OR stand up OR stand ups OR roll*) |
| S194 | TI (chair N3 (stand* OR rise*)) OR AB (chair N3 (stand* OR rise*)) |
| S193 | TI (Berg balance scale OR Tinetti* OR performance oriented mobility assessment* OR tandem walk* OR lower extremity motor coordination test*) OR AB (Berg balance scale OR Tinetti* OR performance oriented mobility assessment* OR tandem walk* OR lower extremity motor coordination test*) |
| S192 | TI (chair N3 (stand* OR rise*)) OR AB (chair N3 (stand* OR rise*)) |
| S191 | TI (clinical test* N2 sensory interaction N2 balance) OR AB (clinical test* N2 sensory interaction N2 balance) |
| S190 | TI (arch-up* OR sit-up* OR squat* OR dumbbell press* OR double limb stance OR single limb stance OR stork stand* OR flamingo balance OR y-balance) OR AB (arch-up* OR sit-up* OR squat* OR dumbbell press* OR double limb stance OR single limb stance OR stork stand* OR flamingo balance OR y-balance) |
| S189 | TI CTSIB OR AB CTSIB |
| S188 | TI ((lower extremities OR (hips AND knees)) N5 (90deg* OR "90 degree*")) OR AB ((lower extremities OR (hips AND knees)) N5 (90deg* OR "90 degree*")) |
| S187 | (MH "One Leg Stand") |
| S186 | TI prone bridge OR AB prone bridge |
| S185 | TI ((back OR body OR trunk) N3 endurance) OR AB ((back OR body OR trunk) N3 endurance) |
| S184 | TI (sternum N3 (ground OR floor)) OR AB (sternum N3 (ground OR floor)) |
| S183 | TI (aerobic capacity OR VO2* OR bicycle ergomet* OR maximal graded exercise* OR steep ramp OR muscle endurance OR Biering Sorensen OR modified Sorensen OR Roman chair) OR AB (aerobic capacity OR VO2* OR bicycle ergomet* OR maximal graded exercise* OR steep ramp OR muscle endurance OR Biering Sorensen OR modified Sorensen OR Roman chair) |
| S182 | (MH "Aerobic Capacity") |
| S181 | S173 OR S174 OR S175 OR S176 OR S177 OR S178 OR S179 OR S180 |
| S180 | TI lying or AB lying |
| S179 | TI (time N5 stand*) OR AB (time N5 stand*) |
| S178 | (MH "Exercise Intensity") |
| S177 | (MH "Accelerometers") OR (MH "Accelerometry") |
| S176 | TI (constant postures OR active postures OR sedentary postures OR sedentary activity OR walking time OR walking distance OR claudication index OR walking speed OR daily walking events OR light intensity OR moderate intensity OR vigorous intensity OR activity count OR gait cycles OR gait posture index OR physical activity OR acceleromet*) OR AB (constant postures OR active postures OR sedentary postures OR sedentary activity OR walking time OR walking distance OR claudication index OR walking speed OR daily walking events OR light intensity OR moderate intensity OR vigorous intensity OR activity count OR gait cycles OR gait posture index OR physical activity OR acceleromet*) |
| S175 | (MH "Fitness Trackers") OR (MH "Pedometers") |
| S174 | TI (activity monitor OR pedomet* OR GPS OR watch OR smartwatch) OR AB (activity monitor OR pedomet* OR GPS OR watch OR smartwatch) |
| S173 | TI (6WT N3 app*) OR AB (6WT N3 app*) |
| S172 | S164 OR S165 OR S166 OR S167 OR S168 OR S169 OR S170 OR S171 |
| S171 | (back* OR lumbosacral OR lumbo-sacral OR lumbar) |
| S170 | (radiculopathy OR radiculitis OR radicular pain*) |
| S169 | (lumbago OR sciatica) |
| S168 | (lumbar OR lumbosacral OR lumbo-sacral OR low* back*) |
| S167 | (lumbar vertebra* OR lumbar spin* OR lumbar disk OR lumbar disc) |
| S166 | "(lumbar vertebra* OR lumbar spin* OR lumbar disk OR lumbar disc)" |
| S165 | (MH "Sciatica") |
| S164 | (MH "Lumbar Vertebrae") |
| S163 | S117 AND S160 AND S161 |
| S161 | S126 OR S159 |
| S160 | TI prognos* or AB prognos* |
| S159 | S127 OR S128 OR S129 OR S130 OR S131 OR S132 OR S133 OR S134 OR S135 OR S136 OR S137 OR S138 OR S139 OR S140 OR S141 OR S142 OR S143 OR S144 OR S145 OR S146 OR S147 OR S148 OR S149 OR S150 OR S151 OR S152 OR S153 OR S154 OR S155 OR S156 OR S157 OR S158 |
| S158 | TI (Physiotherapy functional mobility profile OR Barthel index) OR AB (Physiotherapy functional mobility profile OR Barthel index) |
| S157 | (MH "Barthel Index") |
| S156 | TI Activity measure for post-acute care 6 clicks OR AB Activity measure for post-acute care 6 clicks |
| S155 | TI ("timed up and go" OR TUG OR "8 foot up and go" OR Physical capability assessment tool OR PCAT OR aggregated functional performance test OR aggregated assessment of physical function OR short physical performance battery OR cumulated ambulation score OR functional independence measure OR Katz ADL index) OR AB ("timed up and go" OR TUG OR "8 foot up and go" OR Physical capability assessment tool OR PCAT OR aggregated functional performance test OR aggregated assessment of physical function OR short physical performance battery OR cumulated ambulation score OR functional independence measure OR Katz ADL index) |
| S154 | TI (stair* N2 climb*) OR AB (stair* N2 climb*) |
| S153 | (MH "Stair Climbing") |
| S152 | TI shuttle walk* OR AB shuttle walk* |
| S151 | TI (walk N3 hall*) OR AB (walk N3 hall*) |
| S150 | TI (self-paced walk* OR 4-meter walk* OR 4-metre walk* OR 5-meter walk* OR 5-metre walk* OR 10-meter walk* OR 10-metre walk* OR 15-meter walk* OR 15-metre walk* OR 50-meter walk* OR 50-metre walk* OR 50-foot walk* OR 5-minute walk* OR 6-minute walk* OR treadmill OR overground walk*) OR AB (self-paced walk* OR 4-meter walk* OR 4-metre walk* OR 5-meter walk* OR 5-metre walk* OR 10-meter walk* OR 10-metre walk* OR 15-meter walk* OR 15-metre walk* OR 50-meter walk* OR 50-metre walk* OR 50-foot walk* OR 5-minute walk* OR 6-minute walk* OR treadmill OR overground walk*) |
| S149 | TI (lift* OR progressive isoinertial lifting evaluation OR pile OR forward reach OR functional reach) OR AB (lift* OR progressive isoinertial lifting evaluation OR pile OR forward reach OR functional reach) |
| S148 | (MH "Reaching") |
| S147 | (MH "Lifting") |
| S146 | TI functional capacity evaluation OR AB functional capacity evaluation |
| S145 | TI ((stand OR standing) N2 continuous) OR AB (stand OR standing) N2 continuous) |
| S144 | TI step* OR AB step* |
| S143 | (MH "Step") |
| S142 | TI (bed N2 chair) OR AB (bed N2 chair) |
| S141 | TI (lie N2 sit) OR AB (lie N2 sit) |
| S140 | TI (sit to stand OR stand up OR stand ups OR roll*) OR AB (sit to stand OR stand up OR stand ups OR roll*) |
| S139 | TI (chair N3 (stand* OR rise*)) OR AB (chair N3 (stand* OR rise*)) |
| S138 | TI (Berg balance scale OR Tinetti* OR performance oriented mobility assessment* OR tandem walk* OR lower extremity motor coordination test*) OR AB (Berg balance scale OR Tinetti* OR performance oriented mobility assessment* OR tandem walk* OR lower extremity motor coordination test*) |
| S137 | TI (chair N3 (stand* OR rise*)) OR AB (chair N3 (stand* OR rise*)) |
| S136 | TI (clinical test* N2 sensory interaction N2 balance) OR AB (clinical test* N2 sensory interaction N2 balance) |
| S135 | TI (arch-up* OR sit-up* OR squat* OR dumbbell press* OR double limb stance OR single limb stance OR stork stand* OR flamingo balance OR y-balance) OR AB (arch-up* OR sit-up* OR squat* OR dumbbell press* OR double limb stance OR single limb stance OR stork stand* OR flamingo balance OR y-balance) |
| S134 | TI CTSIB OR AB CTSIB |
| S133 | TI ((lower extremities OR (hips AND knees)) N5 (90deg* OR "90 degree*")) OR AB ((lower extremities OR (hips AND knees)) N5 (90deg* OR "90 degree*")) |
| S132 | (MH "One Leg Stand") |
| S131 | TI prone bridge OR AB prone bridge |
| S130 | TI ((back OR body OR trunk) N3 endurance) OR AB ((back OR body OR trunk) N3 endurance) |
| S129 | TI (sternum N3 (ground OR floor)) OR AB (sternum N3 (ground OR floor)) |
| S128 | TI (aerobic capacity OR VO2* OR bicycle ergomet* OR maximal graded exercise* OR steep ramp OR muscle endurance OR Biering Sorensen OR modified Sorensen OR Roman chair) OR AB (aerobic capacity OR VO2* OR bicycle ergomet* OR maximal graded exercise* OR steep ramp OR muscle endurance OR Biering Sorensen OR modified Sorensen OR Roman chair) |
| S127 | (MH "Aerobic Capacity") |
| S126 | S118 OR S119 OR S120 OR S121 OR S122 OR S123 OR S124 OR S125 |
| S125 | TI lying or AB lying |
| S124 | TI (time N5 stand*) OR AB (time N5 stand*) |
| S123 | (MH "Exercise Intensity") |
| S122 | (MH "Accelerometers") OR (MH "Accelerometry") |
| S121 | TI (constant postures OR active postures OR sedentary postures OR sedentary activity OR walking time OR walking distance OR claudication index OR walking speed OR daily walking events OR light intensity OR moderate intensity OR vigorous intensity OR activity count OR gait cycles OR gait posture index OR physical activity OR acceleromet*) OR AB (constant postures OR active postures OR sedentary postures OR sedentary activity OR walking time OR walking distance OR claudication index OR walking speed OR daily walking events OR light intensity OR moderate intensity OR vigorous intensity OR activity count OR gait cycles OR gait posture index OR physical activity OR acceleromet*) |
| S120 | (MH "Fitness Trackers") OR (MH "Pedometers") |
| S119 | TI (activity monitor OR pedomet* OR GPS OR watch OR smartwatch) OR AB (activity monitor OR pedomet* OR GPS OR watch OR smartwatch) |
| S118 | TI (6WT N3 app*) OR AB (6WT N3 app*) |
| S117 | S109 OR S110 OR S111 OR S112 OR S113 OR S114 OR S115 OR S116 |
| S116 | (back* OR lumbosacral OR lumbo-sacral OR lumbar) |
| S115 | (radiculopathy OR radiculitis OR radicular pain*) |
| S114 | (lumbago OR sciatica) |
| S113 | (lumbar OR lumbosacral OR lumbo-sacral OR low* back*) |
| S112 | (lumbar vertebra* OR lumbar spin* OR lumbar disk OR lumbar disc) |
| S111 | "(lumbar vertebra* OR lumbar spin* OR lumbar disk OR lumbar disc)" |
| S110 | (MH "Sciatica") |
| S109 | (MH "Lumbar Vertebrae") |
| S107 | S72 OR S105 |
| S106 | ((TI prognos* or AB prognos*) or (MH “prospective studies” not MM “prospective studies”)) |
| S105 | S73 OR S74 OR S75 OR S76 OR S77 OR S78 OR S79 OR S80 OR S81 OR S82 OR S83 OR S84 OR S85 OR S86 OR S87 OR S88 OR S89 OR S90 OR S91 OR S92 OR S93 OR S94 OR S95 OR S96 OR S97 OR S98 OR S99 OR S100 OR S101 OR S102 OR S103 OR S104 |
| S104 | TI (Physiotherapy functional mobility profile OR Barthel index) OR AB (Physiotherapy functional mobility profile OR Barthel index) |
| S103 | (MH "Barthel Index") |
| S102 | TI Activity measure for post-acute care 6 clicks OR AB Activity measure for post-acute care 6 clicks |
| S101 | TI ("timed up and go" OR TUG OR "8 foot up and go" OR Physical capability assessment tool OR PCAT OR aggregated functional performance test OR aggregated assessment of physical function OR short physical performance battery OR cumulated ambulation score OR functional independence measure OR Katz ADL index) OR AB ("timed up and go" OR TUG OR "8 foot up and go" OR Physical capability assessment tool OR PCAT OR aggregated functional performance test OR aggregated assessment of physical function OR short physical performance battery OR cumulated ambulation score OR functional independence measure OR Katz ADL index) |
| S100 | TI (stair* N2 climb*) OR AB (stair* N2 climb*) |
| S99 | (MH "Stair Climbing") |
| S98 | TI shuttle walk* OR AB shuttle walk* |
| S97 | TI (walk N3 hall*) OR AB (walk N3 hall*) |
| S96 | TI (self-paced walk* OR 4-meter walk* OR 4-metre walk* OR 5-meter walk* OR 5-metre walk* OR 10-meter walk* OR 10-metre walk* OR 15-meter walk* OR 15-metre walk* OR 50-meter walk* OR 50-metre walk* OR 50-foot walk* OR 5-minute walk* OR 6-minute walk* OR treadmill OR overground walk*) OR AB (self-paced walk* OR 4-meter walk* OR 4-metre walk* OR 5-meter walk* OR 5-metre walk* OR 10-meter walk* OR 10-metre walk* OR 15-meter walk* OR 15-metre walk* OR 50-meter walk* OR 50-metre walk* OR 50-foot walk* OR 5-minute walk* OR 6-minute walk* OR treadmill OR overground walk*) |
| S95 | TI (lift* OR progressive isoinertial lifting evaluation OR pile OR forward reach OR functional reach) OR AB (lift* OR progressive isoinertial lifting evaluation OR pile OR forward reach OR functional reach) |
| S94 | (MH "Reaching") |
| S93 | (MH "Lifting") |
| S92 | TI functional capacity evaluation OR AB functional capacity evaluation |
| S91 | TI ((stand OR standing) N2 continuous) OR AB (stand OR standing) N2 continuous) |
| S90 | TI step* OR AB step* |
| S89 | (MH "Step") |
| S88 | TI (bed N2 chair) OR AB (bed N2 chair) |
| S87 | TI (lie N2 sit) OR AB (lie N2 sit) |
| S86 | TI (sit to stand OR stand up OR stand ups OR roll*) OR AB (sit to stand OR stand up OR stand ups OR roll*) |
| S85 | TI (chair N3 (stand* OR rise*)) OR AB (chair N3 (stand* OR rise*)) |
| S84 | TI (Berg balance scale OR Tinetti* OR performance oriented mobility assessment* OR tandem walk* OR lower extremity motor coordination test*) OR AB (Berg balance scale OR Tinetti* OR performance oriented mobility assessment* OR tandem walk* OR lower extremity motor coordination test*) |
| S83 | TI (chair N3 (stand* OR rise*)) OR AB (chair N3 (stand* OR rise*)) |
| S82 | TI (clinical test* N2 sensory interaction N2 balance) OR AB (clinical test* N2 sensory interaction N2 balance) |
| S81 | TI (arch-up* OR sit-up* OR squat* OR dumbbell press* OR double limb stance OR single limb stance OR stork stand* OR flamingo balance OR y-balance) OR AB (arch-up* OR sit-up* OR squat* OR dumbbell press* OR double limb stance OR single limb stance OR stork stand* OR flamingo balance OR y-balance) |
| S80 | TI CTSIB OR AB CTSIB |
| S79 | TI ((lower extremities OR (hips AND knees)) N5 (90deg* OR "90 degree*")) OR AB ((lower extremities OR (hips AND knees)) N5 (90deg* OR "90 degree*")) |
| S78 | (MH "One Leg Stand") |
| S77 | TI prone bridge OR AB prone bridge |
| S76 | TI ((back OR body OR trunk) N3 endurance) OR AB ((back OR body OR trunk) N3 endurance) |
| S75 | TI (sternum N3 (ground OR floor)) OR AB (sternum N3 (ground OR floor)) |
| S74 | TI (aerobic capacity OR VO2* OR bicycle ergomet* OR maximal graded exercise* OR steep ramp OR muscle endurance OR Biering Sorensen OR modified Sorensen OR Roman chair) OR AB (aerobic capacity OR VO2* OR bicycle ergomet* OR maximal graded exercise* OR steep ramp OR muscle endurance OR Biering Sorensen OR modified Sorensen OR Roman chair) |
| S73 | (MH "Aerobic Capacity") |
| S72 | S64 OR S65 OR S66 OR S67 OR S68 OR S69 OR S70 OR S71 |
| S71 | TI lying or AB lying |
| S70 | TI (time N5 stand*) OR AB (time N5 stand*) |
| S69 | (MH "Exercise Intensity") |
| S68 | (MH "Accelerometers") OR (MH "Accelerometry") |
| S67 | TI (constant postures OR active postures OR sedentary postures OR sedentary activity OR walking time OR walking distance OR claudication index OR walking speed OR daily walking events OR light intensity OR moderate intensity OR vigorous intensity OR activity count OR gait cycles OR gait posture index OR physical activity OR acceleromet*) OR AB (constant postures OR active postures OR sedentary postures OR sedentary activity OR walking time OR walking distance OR claudication index OR walking speed OR daily walking events OR light intensity OR moderate intensity OR vigorous intensity OR activity count OR gait cycles OR gait posture index OR physical activity OR acceleromet*) |
| S66 | (MH "Fitness Trackers") OR (MH "Pedometers") |
| S65 | TI (activity monitor OR pedomet* OR GPS OR watch OR smartwatch) OR AB (activity monitor OR pedomet* OR GPS OR watch OR smartwatch) |
| S64 | TI (6WT N3 app*) OR AB (6WT N3 app*) |
| S63 | S55 OR S56 OR S57 OR S58 OR S59 OR S60 OR S61 OR S62 |
| S62 | (back* OR lumbosacral OR lumbo-sacral OR lumbar) |
| S61 | (radiculopathy OR radiculitis OR radicular pain*) |
| S60 | (lumbago OR sciatica) |
| S59 | (lumbar OR lumbosacral OR lumbo-sacral OR low* back*) |
| S58 | (lumbar vertebra* OR lumbar spin* OR lumbar disk OR lumbar disc) |
| S57 | "(lumbar vertebra* OR lumbar spin* OR lumbar disk OR lumbar disc)" |
| S56 | (MH "Sciatica") |
| S55 | (MH "Lumbar Vertebrae") |
| S54 | S9 AND S52 AND S53 |
| S53 | S18 OR S51 |
| S52 | ((TI prognos* or AB prognos*) or (MH “prospective studies” not MM “prospective studies”)) |
| S51 | S19 OR S20 OR S21 OR S22 OR S23 OR S24 OR S25 OR S26 OR S27 OR S28 OR S29 OR S30 OR S31 OR S32 OR S33 OR S34 OR S35 OR S36 OR S37 OR S38 OR S39 OR S40 OR S41 OR S42 OR S43 OR S44 OR S45 OR S46 OR S47 OR S48 OR S49 OR S50 |
| S50 | TI (Physiotherapy functional mobility profile OR Barthel index) OR AB (Physiotherapy functional mobility profile OR Barthel index) |
| S49 | (MH "Barthel Index") |
| S48 | TI Activity measure for post-acute care 6 clicks OR AB Activity measure for post-acute care 6 clicks |
| S47 | TI ("timed up and go" OR TUG OR "8 foot up and go" OR Physical capability assessment tool OR PCAT OR aggregated functional performance test OR aggregated assessment of physical function OR short physical performance battery OR cumulated ambulation score OR functional independence measure OR Katz ADL index) OR AB ("timed up and go" OR TUG OR "8 foot up and go" OR Physical capability assessment tool OR PCAT OR aggregated functional performance test OR aggregated assessment of physical function OR short physical performance battery OR cumulated ambulation score OR functional independence measure OR Katz ADL index) |
| S46 | TI (stair* N2 climb*) OR AB (stair* N2 climb*) |
| S45 | (MH "Stair Climbing") |
| S44 | TI shuttle walk* OR AB shuttle walk* |
| S43 | TI (walk N3 hall*) OR AB (walk N3 hall*) |
| S42 | TI (self-paced walk* OR 4-meter walk* OR 4-metre walk* OR 5-meter walk* OR 5-metre walk* OR 10-meter walk* OR 10-metre walk* OR 15-meter walk* OR 15-metre walk* OR 50-meter walk* OR 50-metre walk* OR 50-foot walk* OR 5-minute walk* OR 6-minute walk* OR treadmill OR overground walk*) OR AB (self-paced walk* OR 4-meter walk* OR 4-metre walk* OR 5-meter walk* OR 5-metre walk* OR 10-meter walk* OR 10-metre walk* OR 15-meter walk* OR 15-metre walk* OR 50-meter walk* OR 50-metre walk* OR 50-foot walk* OR 5-minute walk* OR 6-minute walk* OR treadmill OR overground walk*) |
| S41 | TI (lift* OR progressive isoinertial lifting evaluation OR pile OR forward reach OR functional reach) OR AB (lift* OR progressive isoinertial lifting evaluation OR pile OR forward reach OR functional reach) |
| S40 | (MH "Reaching") |
| S39 | (MH "Lifting") |
| S38 | TI functional capacity evaluation OR AB functional capacity evaluation |
| S37 | TI ((stand OR standing) N2 continuous) OR AB (stand OR standing) N2 continuous) |
| S36 | TI step* OR AB step* |
| S35 | (MH "Step") |
| S34 | TI (bed N2 chair) OR AB (bed N2 chair) |
| S33 | TI (lie N2 sit) OR AB (lie N2 sit) |
| S32 | TI (sit to stand OR stand up OR stand ups OR roll*) OR AB (sit to stand OR stand up OR stand ups OR roll*) |
| S31 | TI (chair N3 (stand* OR rise*)) OR AB (chair N3 (stand* OR rise*)) |
| S30 | TI (Berg balance scale OR Tinetti* OR performance oriented mobility assessment* OR tandem walk* OR lower extremity motor coordination test*) OR AB (Berg balance scale OR Tinetti* OR performance oriented mobility assessment* OR tandem walk* OR lower extremity motor coordination test*) |
| S29 | TI (chair N3 (stand* OR rise*)) OR AB (chair N3 (stand* OR rise*)) |
| S28 | TI (clinical test* N2 sensory interaction N2 balance) OR AB (clinical test* N2 sensory interaction N2 balance) |
| S27 | TI (arch-up* OR sit-up* OR squat* OR dumbbell press* OR double limb stance OR single limb stance OR stork stand* OR flamingo balance OR y-balance) OR AB (arch-up* OR sit-up* OR squat* OR dumbbell press* OR double limb stance OR single limb stance OR stork stand* OR flamingo balance OR y-balance) |
| S26 | TI CTSIB OR AB CTSIB |
| S25 | TI ((lower extremities OR (hips AND knees)) N5 (90deg* OR "90 degree*")) OR AB ((lower extremities OR (hips AND knees)) N5 (90deg* OR "90 degree*")) |
| S24 | (MH "One Leg Stand") |
| S23 | TI prone bridge OR AB prone bridge |
| S22 | TI ((back OR body OR trunk) N3 endurance) OR AB ((back OR body OR trunk) N3 endurance) |
| S21 | TI (sternum N3 (ground OR floor)) OR AB (sternum N3 (ground OR floor)) |
| S20 | TI (aerobic capacity OR VO2* OR bicycle ergomet* OR maximal graded exercise* OR steep ramp OR muscle endurance OR Biering Sorensen OR modified Sorensen OR Roman chair) OR AB (aerobic capacity OR VO2* OR bicycle ergomet* OR maximal graded exercise* OR steep ramp OR muscle endurance OR Biering Sorensen OR modified Sorensen OR Roman chair) |
| S19 | (MH "Aerobic Capacity") |
| S18 | S10 OR S11 OR S12 OR S13 OR S14 OR S15 OR S16 OR S17 |
| S17 | TI lying or AB lying |
| S16 | TI (time N5 stand*) OR AB (time N5 stand*) |
| S15 | (MH "Exercise Intensity") |
| S14 | (MH "Accelerometers") OR (MH "Accelerometry") |
| S13 | TI (constant postures OR active postures OR sedentary postures OR sedentary activity OR walking time OR walking distance OR claudication index OR walking speed OR daily walking events OR light intensity OR moderate intensity OR vigorous intensity OR activity count OR gait cycles OR gait posture index OR physical activity OR acceleromet*) OR AB (constant postures OR active postures OR sedentary postures OR sedentary activity OR walking time OR walking distance OR claudication index OR walking speed OR daily walking events OR light intensity OR moderate intensity OR vigorous intensity OR activity count OR gait cycles OR gait posture index OR physical activity OR acceleromet*) |
| S12 | (MH "Fitness Trackers") OR (MH "Pedometers") |
| S11 | TI (activity monitor OR pedomet* OR GPS OR watch OR smartwatch) OR AB (activity monitor OR pedomet* OR GPS OR watch OR smartwatch) |
| S10 | TI (6WT N3 app*) OR AB (6WT N3 app*) |
| S9 | S1 OR S2 OR S3 OR S4 OR S5 OR S6 OR S7 OR S8 |
| S8 | (back* OR lumbosacral OR lumbo-sacral OR lumbar) |
| S7 | (radiculopathy OR radiculitis OR radicular pain*) |
| S6 | (lumbago OR sciatica) |
| S5 | (lumbar OR lumbosacral OR lumbo-sacral OR low* back*) |
| S4 | (lumbar vertebra* OR lumbar spin* OR lumbar disk OR lumbar disc) |
| S3 | "(lumbar vertebra* OR lumbar spin* OR lumbar disk OR lumbar disc)" |
| S2 | (MH "Sciatica") |
| S1 | (MH "Lumbar Vertebrae") |

**Scopus**1947 to May 29, 2024 N= 4159
Search Strategy:

(TITLE-ABS-KEY(lumbar OR lumbosacral OR "lumbo-sacral" OR "low* back*") OR TITLE-ABS-KEY(backpain OR backache) OR TITLE-ABS-KEY (sciatica OR radiculopathy OR radiculitis OR "radicular pain")) AND ((TITLE-ABS-KEY("range of motion" OR ROM) OR TITLE-ABS-KEY(finger* W/3 (floor OR toe OR knee OR "fibular head")) OR TITLE-ABS-KEY(wrist crease W/3 floor) OR TITLE-ABS-KEY(Schober OR "straight leg raise*" OR SLR OR Lasegue* OR "isometric strength" OR "isokinetic strength" OR "motor control" OR "movement control" OR "functional movement screen*" OR FMS OR "sitting one leg knee extension" OR "posterior pelvic tilt" OR "waiter's bow" OR "one leg stance") OR TITLE-ABS-KEY("gait analysis" OR "spatiotemporal gait" OR "spatio temporal gait" OR "stride length" OR "stride duration" OR "walking speed" OR "gait speed" OR cadence OR "gait asymmetry" OR "stance phase" OR "swing phase" OR "double limb support" OR "single limb support" OR inclinometer OR goniometer OR kyphometer OR "electromagnetic tracking") OR TITLE-ABS-KEY(dynamometer OR "manual muscle test" OR MedX OR Cybex OR Kin-Com OR RehaGait OR JAMAR) OR TITLE-ABS-KEY("aerobic capacity" OR VO2* OR "bicycle ergomet*" OR "maximal graded exercise*" OR "steep ramp" OR "muscle endurance" OR "Biering Sorensen" OR "modified Sorensen" OR "Roman chair") OR TITLE-ABS-KEY(((back OR body OR trunk) W/3 endurance)) OR TITLE-ABS-KEY("prone bridge") OR TITLE-ABS-KEY ("lower extremities" OR (hips AND knees)) W/5 (90deg* OR "90 degree*")) OR TITLE-ABS-KEY(arch-up* OR sit-up* OR squat* OR "dumbbell press*" OR "double limb stance" OR "single limb stance" OR "stork stand*" OR "flamingo balance" OR y-balance OR CTSIB) OR TITLE-ABS-KEY("clinical test*" W/2 "sensory interaction" W/2 balance) OR ("clinical test*" W/2 "sensory integration" W/2 balance) OR TITLE-ABS-KEY("Berg balance scale" OR Tinetti* OR "performance oriented mobility assessment*" OR "tandem walk*" OR "lower extremity motor coordination test*") OR TITLE-ABS-KEY(chair W/3 (stand* OR rise*)) OR TITLE-ABS-KEY("sit to stand" OR "stand up" OR "stand ups" OR roll*) OR TITLE-ABS-KEY(lie W/2 sit) OR TITLE-ABS-KEY(bed W/2 chair) OR TITLE-ABS-KEY(step*) OR TITLE-ABS-KEY(stand or standing) OR TITLE-ABS-KEY("functional capacity evaluation" OR lift* OR "progressive isoinertial lifting evaluation" OR pile OR "forward reach" OR "functional reach") OR TITLE-ABS-KEY("walk test*" OR "self-paced walk*" OR "4-meter walk*" OR "4-metre walk*" OR "5-meter walk*" OR "5-metre walk*" OR "10-meter walk*" OR "10-metre walk*" OR "15-meter walk*" OR "15-metre walk*" OR "50-meter walk*" OR "50-metre walk*" OR "50-foot walk*" OR "5-minute walk*" OR "6-minute walk*" OR treadmill OR "overground walk*" OR "shuttle walk") OR TITLE-ABS-KEY(walk W/3 hall*) OR TITLE-ABS-KEY(stair* W/2 climb*) OR TITLE-ABS-KEY("timed up and go" OR TUG OR "8 foot up and go" OR "Physical capability assessment tool" OR PCAT OR "aggregated functional performance test" OR "aggregated assessment of physical function" OR "short physical performance battery" OR "cumulated ambulation score" OR "functional independence measure" OR "Katz ADL index" OR "Katz index") OR TITLE-ABS-KEY("Activity measure for post-acute care 6 clicks" OR "Activity measure for postacute care 6 clicks" OR "Physiotherapy functional mobility profile" OR "Barthel index") OR TITLE-ABS-KEY(lying) OR TITLE-ABS-KEY(time W/5 stand*) OR TITLE-ABS-KEY("constant postures" OR "active postures" OR "sedentary postures" OR "sedentary activity" OR "walking time" OR "walking distance" OR "claudication index" OR "daily walking events" OR "light intensity" OR "moderate intensity" OR "vigorous intensity" OR "activity count" OR "gait cycles" OR "gait posture index" OR "physical activity" OR acceleromet*) OR TITLE-ABS-KEY("wearable electronic devices" OR "fitness tracker*" OR "activity monitor*" OR pedomet* OR gps OR watch OR "smart watch") OR TITLE-ABS-KEY(6wt W/3 app*)) AND TITLE-ABS-KEY(prognos* OR predict* OR "causal factor" OR risk*)

**ProQuest Dissertations & Theses run May 29, 2024 – N=627**

(((((noft("low* back pain*") OR noft("low* back ache*") OR noft("backache* low*") OR noft("backpain* low*") OR noft("lumbar") OR noft("lumbosacral") OR noft("lumbo-sacral") OR noft("lumbago") OR noft("sciatica") OR noft("radiculopathy") OR noft("radiculitis") OR noft("radicular pain*") OR noft("spinal pain") OR noft("vertebral pain") OR noft("chronic back pain") OR noft("degenerative spine conditions") OR noft("chronic low back pain") OR noft("mechanical low back pain") OR noft("discogenic pain") OR noft("nerve root pain") OR noft("facet joint pain") OR noft("lumbar disc herniation") OR noft("lumbar degenerative disease") OR noft("spondylosis") OR noft("lumbar instability"))) AND (noft("physical outcome measur*") OR noft("physical measures of function*") OR noft("measures of function*") OR noft("physical function* test") OR noft("functional assessment") OR noft("range of motion") OR noft("ROM") OR noft("Schober") OR noft("finger* NEAR/3 (floor OR toe OR knee OR fibular head)") OR noft("wrist crease NEAR/3 floor") OR noft("straight leg raise*") OR noft("SLR") OR noft("Lasegue*") OR noft("isometric strength") OR noft("isokinetic strength") OR noft("motor control") OR noft("movement control") OR noft("functional movement screen*") OR noft("functional tests") OR noft("mobility assessment") OR noft("physical performance evaluation") OR noft("functional capacity tests") OR noft("gait analysis") OR noft("walking analysis") OR noft("inclinometer") OR noft("goniometer") OR noft("kyphometer") OR noft("dynamometer") OR noft("manual muscle test") OR noft("MedX") OR noft("Cybex") OR noft("Kin-Com") OR noft("RehaGait") OR noft("JAMAR") OR noft("aerobic capacity") OR noft("VO2*") OR noft("bicycle ergomet*") OR noft("maximal graded exercise*") OR noft("steep ramp") OR noft("muscle endurance") OR noft("Biering Sorensen") OR noft("modified Sorensen") OR noft("prone bridge") OR noft("lower extremities NEAR/5 (90deg* OR 90 degree*)") OR noft("arch-up*") OR noft("sit-up*") OR noft("squat*") OR noft("6-minute walk test") OR noft("10-meter walk test") OR noft("shuttle walk*") OR noft("step*") OR noft("stair* climb*") OR noft("timed up and go") OR noft("TUG") OR noft("activity monitor") OR noft("acceleromet*") OR noft("fitness tracker") OR noft("wearable electronic device") OR noft("smartwatch") OR noft("GPS") OR noft("physical performance test") OR noft("functional mobility") OR noft("physical activity") OR noft("functional capacity evaluation") OR noft("performance measures") OR noft("balance assessment") OR noft("motor coordination tests") OR noft("functional ability"))) AND (noft("risk") OR noft("cohort") OR noft("prognos*") OR noft("predict*") OR noft("incidence") OR noft("survival analysis") OR noft("causal factor") OR noft("course") OR noft("outcomes") OR noft("determinants") OR noft("predictive factors") OR noft("risk factors") OR noft("recovery trajectory") OR noft("prognostic indicators") OR noft("prognostic outcomes") OR noft("outcome prediction") OR noft("recovery factors") OR noft("predictive indicators") OR noft("prognostic models"))))
