# Supplementary material for: Physical measures of physical functioning as prognostic factors to predict outcomes in low back pain: A systematic review and narrative synthesis
Source: PLoS One. 2025 Oct 28;20(10):e0335535. doi: 10.1371/journal.pone.0335535 (PMC12561921; doi:10.1371/journal.pone.0335535)
Supplement: S3 File — (DOCX) [file pone.0335535.s003.docx]

**Quality in Prognostic Studies (QUIPS) Domains for Risk of Bias Assessment**

**Study Participation:**

- The study sample adequately represents the population of interest.
- Adequate participation in the study by eligible persons.
- Description of the source population or population of interest.
- Description of the baseline study sample.
- Adequate description of the sampling frame and recruitment.
- Adequate description of the period and place of recruitment.
- Adequate description of inclusion and exclusion criteria.

**Study Attrition:**

- The study data available (i.e., participants not lost to follow-up) adequately represent the study sample.
- Adequate response rate for study participants.
- Description of attempts to collect information on participants who dropped out.
- Reasons for loss to follow-up are provided.
- Adequate description of participants lost to follow-up.
- No important differences between participants who completed the study and those who did not.

**Prognostic Factor Measurement:**

- The PF is measured in a similar way for all participants.
- A clear definition or description of the PF is provided.
- The method of PF measurement is adequately valid and reliable.
- Continuous variables are reported, or appropriate cut points are used.
- The method and setting of measurement of the PF are the same for all study participants.
- An adequate proportion of the study sample has complete data for the PF.
- Appropriate methods of imputation are used for missing PF data.

**Outcome Measurement:**

- The outcome of interest is measured similarly for all participants.
- A clear definition of the outcome is provided.
- The method of outcome measurement is adequately valid and reliable.
- The method and setting of outcome measurement are the same for all study participants.

**Study Confounding:**

- Important potential confounding factors are appropriately accounted for.
- All important confounders are measured.
- Clear definitions of the important confounders measured are provided.
- The measurement of all important confounders is adequately valid and reliable.
- The method and setting of confounding measurement are the same for all study participants.
- Appropriate methods are used if imputation is applied for missing confounder data.
- Important potential confounders are accounted for in the study design.
- Important potential confounders are accounted for in the analysis.

**Statistical Analysis and Reporting:**

- The statistical analysis is appropriate, and all primary outcomes are reported.
- Sufficient presentation of data to assess the adequacy of the analytic strategy.
- The strategy for model building is appropriate and based on a conceptual framework or model.
- The selected statistical model is adequate for the design of the study.
- There is no selective reporting of results.

**Quality in Prognostic Studies (QUIPS) Tool for Risk of Bias Assessment**

| Domain | Low Risk of Bias | Moderate Risk of Bias | High Risk of Bias |
| --- | --- | --- | --- |
| Study Participation | The study sample fully represents the population of interest; clear and adequate descriptions of recruitment and baseline sample. | Some selection bias or underrepresentation of subgroups; minor issues with sampling or recruitment descriptions. | Significant selection bias; unclear or inadequate descriptions of recruitment or the baseline sample. |
| Study Attrition | <10% loss to follow-up; reasons for attrition well-documented; minimal differences between completers and non-completers. | 10-20% loss to follow-up; partially explained reasons for attrition; minor differences between groups. | >20% loss to follow-up; unclear or unreported reasons for attrition; significant differences between groups. |
| Prognostic Factor Measurement | PF is measured uniformly across all participants; methods are valid and reliable with complete data or appropriate imputation. | Some variability in PF measurement or minor issues with validity, reliability, or data completeness. | PF measurement varies significantly between participants; poor validity/reliability or incomplete data without proper imputation. |
| Outcome Measurement | Outcomes are measured uniformly and consistently across all participants using valid and reliable methods. | Minor deviations from standard measurement protocols or minor flaws in measurement tools. | Outcomes are measured inconsistently or with invalid/unreliable methods. |
| Study Confounding | All important confounders are measured and adjusted for with clear definitions and consistent measurement across participants. | Some confounders may not be fully accounted for, or there is inconsistency in how confounders are controlled. | Major confounders are not measured or adjusted for, leading to potential distortion of results. |
| Statistical Analysis and Reporting | Statistical analysis is appropriate, fully reported, and based on a strong conceptual framework with no selective reporting. | Mostly appropriate analysis but with minor issues such as incomplete reporting | Inappropriate or poorly reported statistical methods. |

**QUIPS Judgment Formula**

An overall RoB score was decided for each study based on the scores of all domains.

- If all six domains are rated as low RoB, or no more than one is rated as moderate ROB, the study is categorized as low RoB.
- If one or more domains are rated as high RoB or ≥ 3 domains are rated as moderate ROB, the study is assessed as high RoB.
- All studies in between are classified as having moderate ROB
