# Supplementary material for: Physical measures of physical functioning as prognostic factors to predict outcomes in low back pain: A systematic review and narrative synthesis
Source: PLoS One. 2025 Oct 28;20(10):e0335535. doi: 10.1371/journal.pone.0335535 (PMC12561921; doi:10.1371/journal.pone.0335535)
Supplement: S4 File — (DOCX) [file pone.0335535.s004.docx]

**GRADE criteria**

**Study Limitations (Risk of Bias):**

- No downgrading if all studies have low risk of bias (ROB).
- Downgrading is applied when at least one study shows moderate or high ROB.

**Inconsistency:**

- No downgrading if findings are consistent across studies (same direction)
- Downgrading if findings across studies are inconsistent, indicating variability in results (different directions)

**Imprecision:**

- Upgrading if there is a large effect size (r > 0.5) with narrow confidence intervals and an adequate sample size.
- Downgrading occurs if confidence intervals are not provided, effect estimates are small, or sample sizes are insufficient.
- Downgrading is also applied when r values fall between 0.2 and 0.5, or when synthesis is unclearly reported or due to inconsistent reporting of confidence intervals across studies.

**Publication Bias:**

- No downgrading if there is no indication of publication bias, such as selective reporting, or if studies are published in reputable, peer-reviewed journals without significant influence from funding.
- Downgrading occurs if sample size, significance of results, or commercialized funding sources suggest a publication bias.
