# Supplementary material for: Physical measures of physical functioning as prognostic factors to predict outcomes in low back pain: A systematic review and narrative synthesis
Source: PLoS One. 2025 Oct 28;20(10):e0335535. doi: 10.1371/journal.pone.0335535 (PMC12561921; doi:10.1371/journal.pone.0335535)
Supplement: S5 File — (DOCX) [file pone.0335535.s005.docx]

**List of Non-English studies= 9**

| 地域在宅高齢者における転倒恐怖感と日常生活活動との関連 | Association between fear of falling and activities of daily living among community-dwelling Japanese older adults |
| --- | --- |
| 腰部脊柱管狭窄症患者における歩行中の脊柱・骨盤 運動と歩行負荷による腰痛の増悪との相関 | Correlation between spinal and pelvic movements during gait and aggravation of low back pain by gait loading in lumbar spinal stenosis patients. |
| Effekte und Prädiktoren des Outcomes in der Rehabilitation muskuloskelettaler Erkrankungen | Effects and predictors of outcome in the rehabilitation of musculoskeletal diseases |
| Évaluation à quatre ans d’un programme de reconditionnement à l’effort pour lombalgie chronique | Evaluation after four years of exercise therapy for chronic low back pain |
| El test de Lasègue postoperatorio como factor pronóstico de los pacientes intervenidos de hernia discal lumbarLasègue's test as prognostic factor for patients under-going lumbar disc surgery | Lasegue's test as prognostic factor for patients undergoing lumbar disc surgery |
| 破裂型腰椎间盘突出症转归预测因素的 Logistic 回归 | Logistic regression analysis on the outcome predictive factors of ruptured lumbar disc herniation |
| Gebelikte Görülen Bel Ağrıları | Low back pain in pregnancy |
| Rehabilitation von Patienten mit chronischen Rückenbeschwerden: Assessment, Ergebnis und prädiktive Faktoren | Rehabilitation of patients with chronic back pain: Assessment, results and predictive factors - A prospective study with 3 months follow-up |
| STAJYER FİZYOTERAPİSTLERDE NONSPESİFİK BEL AĞRISININ POSTÜR, KOR ENDURANS VE LUMBOPELVİK STABİLİTE İLE İLİŞKİSİ | THE RELATIONSHIP OF NONSPECIFIC LOW BACK PAIN WITH POSTURE, CORE ENDURANCE AND LUMBOPELVIC STABILITY IN INTERN PHYSIOTHERAPISTS |
