# Supplementary material for: Physical measures of physical functioning as prognostic factors to predict outcomes in low back pain: A systematic review and narrative synthesis
Source: PLoS One. 2025 Oct 28;20(10):e0335535. doi: 10.1371/journal.pone.0335535 (PMC12561921; doi:10.1371/journal.pone.0335535)
Supplement: S6 File — (DOCX) [file pone.0335535.s006.docx]

| **S3 File: Reasons of excluded studies at full text screening stage** | | |
| --- | --- | --- |
| **Author** | **Journal** | **Reason** |
| Azimi et al. | Journal of Neurosurgery: Spine | Study design |
| Knox et al. | Journal of Geriatric Physical Therapy | Study design |
| Hassan | ProQuest Dissertations and Theses | Not physical functioning prognostic factors |
| Rabin | PQDT - Global | Study design |
| Childs J.D. et al. | Annals of Internal Medicine | Study design |
| Rabin et al. | The Journal of orthopaedic and sports physical therapy | Aim of study is not relevant |
| Minetama M. et al. | Journal of Orthopaedic Science | Aim of study is not relevant |
| Cheng et al. | Journal of clinical neuroscience | Aim of study is not relevant |
| Veronese et al. | Journals of Gerontology Series A: Biological Sciences & Medical Sciences | Patient population |
| Lee et al. | Journal of the American Geriatrics Society | Not physical functioning prognostic factors |
| Tomkins et al. | Spine | Aim of study is not relevant |
| Chaffin et al. | American Industrial Hygiene Association journal | Study design |
| Li et al. | Artificial Intelligence in Medicine | Not physical functioning prognostic factors |
| Ford et al. | British Journal of Neurosurgery | Not physical functioning prognostic factors |
| Verbunt et al. | European Journal of Pain | Not physical functioning prognostic factors |
| Fritz et al. | Physical Therapy | Study design |
| Russo et al. | Neuromodulation | Aim of study is not relevant |
| Simon et al. | The Clinical journal of pain | Study design |
| Spratt et al. | European spine journal | Not physical functioning prognostic factors |
| Mayer et al. | Pain | Study design |
| Halliday et al. | The Journal of orthopaedic and sports physical therapy | Aim of study is not relevant |
| Knox et al. | The Clinical journal of pain | Aim of study is not relevant |
| Knox et al. | Archives of Physical Medicine and Rehabilitation | Not physical functioning prognostic factors |
| Aubry et al. | North American Spine Society journal | Aim of study is not relevant |
| Carragee et al. | Spine | Not physical functioning prognostic factors |
| Gurcay et al. | Disability and Rehabilitation | Not physical functioning prognostic factors |
| Farrell et al. | The Medical journal of Australia | Study design |
| Rundell et al. | The Spine Journal | Study design |
| Hicks et al. | Clinical Journal of Pain | Not physical functioning prognostic factors |
| Mikkelsson et al. | British Journal of Sports Medicine | Patient population |
| Hicks et al. | Gait & posture | Not physical functioning prognostic factors |
| Walsh | PQDT - UK & Ireland | Study design |
| Hartmann et al. | European spine journal | Aim of study is not relevant |
| Felicio et al. | Spine | Study design |
| Starcevic-Klasan et al. | Collegium antropologicum | Patient population |
| Hunt et al. | Spine | Not physical functioning prognostic factors |
| Just et al. | Journal of Cachexia, Sarcopenia and Muscle | Not physical functioning prognostic factors |
| Dang et al. | iScience | Aim of study is not relevant |
| Thiese | ProQuest Dissertations and Theses | Study design |
| Tomita et al. | Nihon koshu eisei zasshi | Not in English language |
| Kapellusch J.M et al. | Journal of Occupational and Environmental Medicine | Patient population |
| Thomas et al. | Spine | Aim of study is not relevant |
| Gutke et al. | Journal of rehabilitation medicine | Aim of study is not relevant |
| Wada et al. | Journal of clinical neuroscience | Not physical functioning prognostic factors |
| Schaaf S. et al. | PM and R | Not physical functioning prognostic factors |
| Staartjes et al. | Neurosurgical review | Study design |
| Blafoss et al. | Scandinavian journal of work, environment & health | Patient population |
| Demarchi et al. | European Spine Journal | Not physical functioning prognostic factors |
| Sand-Svartrud et al. | Scandinavian Journal of Rheumatology | Not physical functioning prognostic factors |
| Lunde L.-K. et al. | Occupational and Environmental Medicine | Patient population |
| Lunde L.-K. et al. | Scandinavian Journal of Work, Environment and Health | Patient population |
| Filho et al. | American journal of physical medicine & rehabilitation | Study design |
| Lariviere et al. | Clinical biomechanics (Bristol, Avon) | Not physical functioning prognostic factors |
| Vincent et al. | PM & R: the journal of injury, function, and rehabilitation | Study design |
| Kujala et al. | Scandinavian Journal of Medicine & Science in Sports | Patient population |
| KOPPENHAVER et al. | Journal of Orthopaedic & Sports Physical Therapy | Study design |
| Haazen et al. | Journal of Rehabilitation Sciences | Study design |
| Jones M.A. et al. | British Journal of Sports Medicine | Patient population |
| Wang et al. | Computer methods in biomechanics and biomedical engineering | Aim of study is not relevant |
| Helmhout et al. | The Journal of sports medicine and physical fitness | Study design |
| Terrier et al. | Ergonomics | Patient population |
| Bendix et al. | Spine | Study design |
| Staartjes et al. | European spine journal | Aim of study is not relevant |
| Hancock M.J et al. | European Journal of Pain | Aim of study is not relevant |
| Talo S. et al. | Clinical Journal of Pain | Not physical functioning prognostic factors |
| Georgopoulos | PQDT - UK & Ireland | Study design |
| Loni et al. | Journal of Spinal Cord Medicine | Not physical functioning prognostic factors |
| Aoki Y. | Scientific Reports | Study design |
| Tomkins-Lane et al. | Archives of physical medicine and rehabilitation | Aim of study is not relevant |
| Li et al. | World neurosurgery | Aim of study is not relevant |
| Watanabe et al. | International orthopaedics | Aim of study is not relevant |
| Cougot B. et al. | Journal of Occupational Medicine and Toxicology | Not physical functioning prognostic factors |
| Schinhan et al. | Clinical journal of sport medicine : official journal of the Canadian Academy of Sport Medicine | Study design |
| Kawaguchi et al. | The Journal of bone and joint surgery. American volume | Aim of study is not relevant |
| Nam et al. | CiOS Clinics in Orthopedic Surgery | Study design |
| Vela et al. | Journal of Sport Rehabilitation | Aim of study is not relevant |
| Oriuichi T et al. | Journal of Gastroenterology | Patient population |
| de Vos Andersen et al. | BMC Musculoskeletal Disorders | Not physical functioning prognostic factors |
| Nykvist et al. | International journal of rehabilitation research. | Patient population |
| Reigo et al. | Scandinavian journal of primary health care | Aim of study is not relevant |
| Rundell et al. | The Spine Journal | Not physical functioning prognostic factors |
| Lazennec et al. | The spine journal: official journal of the North American Spine Society | Aim of study is not relevant |
| Pimenta L. et al. | SAS Journal | Study design |
| Pena Jimenez D. et al. | European Spine Journal | Not physical functioning prognostic factors |
| Folkins et al. | Ergonomics | Study design |
| Ellingson et al. | Computer methods in biomechanics and biomedical engineering | Aim of study is not relevant |
| Wells-Federman C. et al. | Journal of Musculoskeletal Pain | Aim of study is not relevant |
| Hansen et al. | European Journal of Physiotherapy | Aim of study is not relevant |
| Guyer et al. | Spine | Aim of study is not relevant |
| Wei et al. | Scientific Reports | Study design |
| Rainville et al. | Spine | Aim of study is not relevant |
| Fehrmann et al. | The Clinical Journal of Pain | Study design |
| Xiong et al. | Computational and Mathematical Methods in Medicine | Not physical functioning prognostic factors |
| Dunn et al. | European Journal of Pain | Not physical functioning prognostic factors |
| Kline et al. | Journal of Dance Medicine & Science | Aim of study is not relevant |
| Kumar S. et al. | Journal of Musculoskeletal Research | Not physical functioning prognostic factors |
| Kuwahara et al. | Journal of Orthopaedic Science | Not in English language |
| Croft et al. | Pain | Study design |
| Heidler et al. | BMC Anesthesiology | Aim of study is not relevant |
| Lariviere C. et al. | PLoS ONE | Study design |
| Hodselmans et al. | Journal of rehabilitation medicine | Study design |
| Ikwaunusi et al. | Journal of back and musculoskeletal rehabilitation | Not physical functioning prognostic factors |
| Videman et al. | Spine | Aim of study is not relevant |
| Applegate et al. | JMIR Serious Games | Aim of study is not relevant |
| Ahmad et al. | Neurosurgery | Not physical functioning prognostic factors |
| MÃ¼ller et al. | European Spine Journal | Not physical functioning prognostic factors |
| Ford J.J. et al. | Archives of Physical Medicine and Rehabilitation | Not physical functioning prognostic factors |
| Shirsat | ProQuest Dissertations and Theses 2007 | Not physical functioning prognostic factors |
| Huijnen et al. | European journal of pain (London, England) | Aim of study is not relevant |
| Schwind J. et al. | Journal of Manual and Manipulative Therapy | Aim of study is not relevant |
| Lariviere et al. | BMC musculoskeletal disorders | Not physical functioning prognostic factors |
| Zwambag et al. | Journal of biomechanics | Patient population |
| Garcia A.N. et al. | Journal of Manual and Manipulative Therapy | Not physical functioning prognostic factors |
| Hahne et al. | The Australian journal of physiotherapy | Study design |
| Rissanen et al. | Journal of rehabilitation medicine | Study design |
| Learman et al. | Manual therapy | Study design |
| Milton et al. | Journal of clinical orthopaedics and trauma | Not physical functioning prognostic factors |
| Champain S. et al. | European Journal of Orthopaedic Surgery and Traumatology | Aim of study is not relevant |
| Ogura H. et al. | Physiotherapy (United Kingdom) | Aim of study is not relevant |
| Jain S. et al. | Physiotherapy (United Kingdom) | Aim of study is not relevant |
| Keskin-Aktan et al. | Journal of Sport Rehabilitation | Patient population |
| Pillastrini P. et al. | Applied Ergonomics | Aim of study is not relevant |
| Magalhaes M.O. et al. | Brazilian Journal of Physical Therapy | Aim of study is not relevant |
| Farin et al. | Physikalische Medizin Rehabilitationsmedizin | Not in English language |
| del Pozo-Cruz et al. | Journal of Rehabilitation Medicine | Study design |
| Beurskens A.J. et al. | Spine | Study design |
| Pimenta L. et al. | International Journal of Spine Surgery | Aim of study is not relevant |
| Ardeshiri A. | Neuromodulation | Not physical functioning prognostic factors |
| Mao et al. | Medical Journal of Chinese People's Liberation Army | Not physical functioning prognostic factors |
| Verfaille S. et al. | Annales de Readaptation et de Medecine Physique | Not in English language |
| Trabin | ProQuest Dissertations and Theses | Not physical functioning prognostic factors |
| Amaral D.D.V. et al. | The Journal of orthopaedic and sports physical therapy | Not physical functioning prognostic factors |
| Knox et al. | The journal of pain | Not physical functioning prognostic factors |
| Eklund et al. | European Journal of Pain | Not physical functioning prognostic factors |
| Elfving et al. | Disability and rehabilitation | Not physical functioning prognostic factors |
| Janssen et al. | Archives of physical medicine and rehabilitation | Study design |
| Tsafatsanis et al. | BMC Medical Informatics & Decision Making | Patient population |
| Vigdal et al. | Pain | Not physical functioning prognostic factors |
| Oleske et al. | Archives of Physical Medicine and Rehabilitation | Not physical functioning prognostic factors |
| Imajo et al. | Journal of orthopaedic science : official journal of the Japanese Orthopaedic Association | Not physical functioning prognostic factors |
| Alschuler et al. | Pain | Aim of study is not relevant |
| Fritz et al. | Physical therapy | Not physical functioning prognostic factors |
| Zadro et al. | European Journal of Pain (United Kingdom) | Not physical functioning prognostic factors |
| Dawson et al. | Pain | Not physical functioning prognostic factors |
| Jakobsson et al. | BMC musculoskeletal disorders | Not physical functioning prognostic factors |
| Lazennec et al. | The spine journal: official journal of the North American Spine Society | Aim of study is not relevant |
| Aghayev E. et al. | European Spine Journal | Aim of study is not relevant |
| Roseen | ProQuest Dissertations and Theses | Aim of study is not relevant |
| Wheeler et al. | Spine | Study design |
| Chen | The Iowa orthopaedic journal | Aim of study is not relevant |
| Ruan et al. | Journal of Occupational Rehabilitation | Patient population |
| Przkora et al. | Current pain and headache reports | Aim of study is not relevant |
| Whitehurst et al. | Archives of physical medicine and rehabilitation | Aim of study is not relevant |
| Panikkar S. et al. | British Journal of Neurosurgery | Study design |
| Vincent et al. | American journal of physical medicine & rehabilitation | Aim of study is not relevant |
| Constantinescu et al. | Diagnostics | Not physical functioning prognostic factors |
| Park | ProQuest Dissertations and Theses | Patient population |
| Cavalcanti et al. | Journal of back and musculoskeletal rehabilitation | Aim of study is not relevant |
| Stief et al. | Spine | Aim of study is not relevant |
| Mancuso et al. | Spine | Not physical functioning prognostic factors |
| Knox et al. | Arthritis research & therapy | Not physical functioning prognostic factors |
| Than et al. | Journal of neurosurgery: Spine | Not physical functioning prognostic factors |
| Staartjes et al. | European spine journal : official publication of the European Spine Society, the European Spinal Deformity Society, and the European Section of the Cervical Spine Research Society | Study design |
| Ogura et al. | Journal of Neurosurgery: Spine | Study design |
| Bogaert et al. | Sensors (Basel, Switzerland) | Patient population |
| Hirano et al. | Journal of orthopaedic science : official journal of the Japanese Orthopaedic Association | Aim of study is not relevant |
| Dang et al. | Journal of occupational medicine and toxicology (London, England) | Patient population |
| Zehr et al. | Journal of biomechanics | Not physical functioning prognostic factors |
| Yang et al. | Journal of Pain Research | Study design |
| Spigt MG et al. | Nederlands Tijdschrift Voor Fysiotherapie | Study design |
| Chapon et al. | World neurosurgery | Study design |
| Reinhold et al. | Spine | Aim of study is not relevant |
| Hahnle et al. | SAS journal | Aim of study is not relevant |
| Delpierre Y.A. et al. | Journal of Bodywork and Movement Therapies | Not physical functioning prognostic factors |
| Oliveira CB | Journal of Orthopaedic & Sports Physical Therapy | Study design |
| Huijnen et al. | European journal of pain | Aim of study is not relevant |
| Bayramoglu et al. | American journal of physical medicine & rehabilitation | Aim of study is not relevant |
| Bigos et al. | Spine | Aim of study is not relevant |
| Iglesias-Casarrubios et al. | Neurocirugia | Not in English language |
| Yu et al. | China journal of orthopaedics and traumatology | Not in English language |
| Zhang et al. | Chinese medical journal | Study design |
| Jarvik et al. | The Spine Journal | Not physical functioning prognostic factors |
| Bredow et al. | Orthopaedic Surgery | Aim of study is not relevant |
| Thomas K.J. et al. | Health technology assessment (Winchester, England) | Aim of study is not relevant |
| Nordstoga et al. | Musculoskeletal science & practice | Not physical functioning prognostic factors |
| Ferguson et al. | Journal of occupational rehabilitation | Study design |
| Baklouti et al. | Scandinavian Journal of Pain | Study design |
| Nacir B. et al. | Turkish Journal of Rheumatology | Not in English language |
| Jamison et al. | The Clinical journal of pain | Not physical functioning prognostic factors |
| Westbrook | ProQuest Dissertations and Theses | Patient population |
| Harms et al. | BMC Musculoskeletal Disorders | Not physical functioning prognostic factors |
| Molinari R.W. et al. | Journal of Spinal Disorders and Techniques | Not physical functioning prognostic factors |
| Burnett et al. | Manual Therapy | Study design |
| Arzoglou et al. | Operative Neurosurgery (Hagerstown, Md.) | Aim of study is not relevant |
| Miyazaki et al. | Archives of Gerontology and Geriatrics | Patient population |
| Matheve et al. | European Journal of Pain (London, England) | Patient population |
| Papavero et al. | Neurosurgery | Aim of study is not relevant |
| Marras et al. | Ergonomics | Patient population |
| Pimenta et al. | Journal of Neurosurgery: Spine | Not physical functioning prognostic factors |
| Chung et al. | Journal of Spinal Disorders and Techniques | Aim of study is not relevant |
| Vissers K. | Regional Anesthesia and Pain Medicine | Aim of study is not relevant |
| Engle et al. | Pain medicine (Malden, Mass.) | Study design |
| Ko et al. | Sensors | Patient population |
| Siccoli et al. | Neurosurgical focus | Not physical functioning prognostic factors |
| Bhak et al. | Journal of Pain | Not physical functioning prognostic factors |
| Ryan et al. | Journal of Back and Musculoskeletal Rehabilitation | Aim of study is not relevant |
| Jiang Y.-Q. et al. | Journal of Clinical Neuroscience | Study design |
| Chen et al. | Chinese medical journal | Aim of study is not relevant |
| Camerlingo et al. | Gerontology | Patient population |
| Ackermans et al. | Spine deformity | Patient population |
| Del Din et al. | Experimental aging research | Patient population |
| Unsgaard-Tøndel M. et al. | Physical therapy | Aim of study is not relevant |
| Wernli K. et al. | European journal of pain (London, England) | Aim of study is not relevant |
| Cohen et al. | Regional anesthesia and pain medicine | Not physical functioning prognostic factors |
| Alamam et al. | Spine Journal | Not physical functioning prognostic factors |
| Roche-Leboucher G. et al. | Spine | Study design |
| Sasaki et al. | Journal of Physical Therapy Science | Aim of study is not relevant |
| Cai et al. | The journals of gerontology. Series A, Biological sciences and medical sciences | Study design |
| Stuelcken et al. | Physical therapy in sport : official journal of the Association of Chartered Physiotherapists in Sports Medicine | Patient population |
| Lampl Y. et al. | Stroke | Patient population |
| Taulaniemi A. et al. | BMC Musculoskeletal Disorders | Aim of study is not relevant |
| Magnusson et al. | European Journal of Pain (United Kingdom) | Not physical functioning prognostic factors |
| Huang et al. | BMC musculoskeletal disorders | Aim of study is not relevant |
| Ammendolia C. et al. | Chiropractic and Manual Therapies | Aim of study is not relevant |
| Shamus | ProQuest Dissertations and Theses | Study design |
| Suri P. et al. | Clinical Orthopaedics and Related Research | Not physical functioning prognostic factors |
| Kahanovitz N. et al. | Spine | Aim of study is not relevant |
| Stienen et al. | Scientific reports | Aim of study is not relevant |
| Waddell et al. | Spine | Study design |
| Sorensen et al. | Spine | Not physical functioning prognostic factors |
| Miranda H. et al. | Scandinavian Journal of Work, Environment and Health | Aim of study is not relevant |
| Seidler A. et al. | Occupational and environmental medicine | Study design |
| Jensen et al. | Spine Journal | Not physical functioning prognostic factors |
| Budithi S. et al. | European spine journal | Aim of study is not relevant |
| Tabasi et al. | Sensors | Aim of study is not relevant |
| Seliverstova et al. | Zhurnal Voprosy Neirokhirurgii Imeni N.N. Burdenko | Not physical functioning prognostic factors |
| Kaaria S.-M. et al. | European Journal of Pain | Aim of study is not relevant |
| Maneii | ProQuest Dissertations and Theses | Study design |
| Trost et al. | Pain | Patient population |
| Toosizadeh et al. | Clinical biomechanics (Bristol, Avon) | Aim of study is not relevant |
| Finkelstein et al. | Journal of Neurosurgery: Spine | Not physical functioning prognostic factors |
| Du Bois et al. | The Spine Journal | Not physical functioning prognostic factors |
| Zemkova et al. | American journal of men's health | Patient population |
| Andersen et al. | BMC Musculoskeletal Disorders | Not physical functioning prognostic factors |
| Otsubo et al. | Scientific reports | Not physical functioning prognostic factors |
| Gupta et al. | Journal of occupational rehabilitation | Study design |
| Ledoux et al. | Journal of manipulative and physiological therapeutics | Aim of study is not relevant |
| Carugno et al. | Cadernos de Saude Publica | Patient population |
| Hamberg-van Reenen et al. | Occupational and Environmental Medicine | Patient population |
| Jorgensen et al. | BMC Musculoskeletal Disorders | Patient population |
| Coelho-Junior et al. | Aging Clinical and Experimental Research | Patient population |
| Master et al. | Spine | Study design |
| Marchand et al. | Frontiers in neurology | Study design |
| Curtis et al. | Spine | Aim of study is not relevant |
| Vucetic et al. | Clinical orthopaedics and related research | Aim of study is not relevant |
| Peterson et al. | Journal of geriatric physical therapy | Study design |
| Iversen et al. | Journal of geriatric physical therapy | Study design |
| Ha et al. | Journal of neurosurgery. Spine | Aim of study is not relevant |
| Okuda et al. | Journal of neurosurgery. Spine | Study design |
| Robert et al. | Schweizerische Medizinische Wochenschrift | Full text not found |
| Robert et al. | Schweizerische medizinische Wochenschrift | Not physical functioning prognostic factors |
| Christie et al. | Archives of Physical Medicine and Rehabilitation | Aim of study is not relevant |
| Maribo et al. | European Spine Journal | Aim of study is not relevant |
| Aasa B. et al. | Physiotherapy (United Kingdom) | Full text not found |
| Fritz J.M. et al. | BMC Family Practice | Aim of study is not relevant |
| Gautschi et al. | Acta Neurochirurgica | Aim of study is not relevant |
| Saravagi et al. | Intelligent Automation and Soft Computing | Aim of study is not relevant |
| Wand et al. | Spine | Not physical functioning prognostic factors |
| Royuela et al. | The Spine Journal | Not physical functioning prognostic factors |
| Winter et al. | Clinical neurology and neurosurgery | Not physical functioning prognostic factors |
| Hickey et al. | Journal of occupational rehabilitation | Not physical functioning prognostic factors |
| Rhon et al. | Medicine and science in sports and exercise | Not physical functioning prognostic factors |
| Ahmad et al. | International journal of occupational safety and ergonomics: JOSE | Aim of study is not relevant |
| Richardsen et al. | BMC Pregnancy & Childbirth | Patient population |
| Jensen O.K. | BMC Musculoskeletal Disorders | Study design |
| Jellema P. | British Journal of General Practice | Not physical functioning prognostic factors |
| AKERSON | ProQuest Dissertations and Theses | Not physical functioning prognostic factors |
| Piela C.R. | Work (Reading, Mass.) | Aim of study is not relevant |
| McCracken L.M. | Behaviour Research and Therapy | Not physical functioning prognostic factors |
| Hayashi K. | European Spine Journal | Study design |
| McIlroy S. | European Spine Journal | Not physical functioning prognostic factors |
| Inose H. | Spine | Study design |
| Enthoven P. | Pain | Not physical functioning prognostic factors |
| Atarod M. | International Journal of Occupational Safety and Ergonomics | Not physical functioning prognostic factors |
| Lydell et al. | Journal of Rehabilitation Medicine | Patient population |
| Le Gall et al. | Archives des Maladies Professionnelles et de Medecine du Travail | Not physical functioning prognostic factors |
| Smyth et al. | International journal of occupational safety and ergonomics | Aim of study is not relevant |
| Arora et al. | The spine journal : official journal of the North American Spine Society | Study design |
| Kosteljanetz et al. | Acta Neurochirurgica | Not physical functioning prognostic factors |
| Le Heron et al. | Journal des Maladies Vasculaires | Not physical functioning prognostic factors |
| Klukowska et al. | European Spine Journal | Not physical functioning prognostic factors |
| Volpato et al. | Journals of Gerontology Series A: Biological Sciences and Medical Sciences | Patient population |
| Ahlaug et al. | The Spine Journal | Study design |
| Karstens et al. | PLoS ONE | Not physical functioning prognostic factors |
| Haldorsen et al. | European Journal of Pain | Study design |
| Hulens et al. | Scandinavian Journal of Medicine & Science in Sports | Patient population |
| Pieber et al. | European Spine Journal | Not physical functioning prognostic factors |
| Hebert et al. | European Spine Journal | Not physical functioning prognostic factors |
| Hebert et al. | Disability and Rehabilitation | Aim of study is not relevant |
| Macfarlane et al. | Pain | Not physical functioning prognostic factors |
| McGirt et al. | Journal of Neurosurgery: Spine | Not physical functioning prognostic factors |
| Klukowska et al. | Acta Neurochirurgica | Aim of study is not relevant |
| Baillie et al. | Journal of Bodywork and Movement Therapies | Not physical functioning prognostic factors |
| Coric et al. | Journal of Neurosurgery: Spine | Study design |
| Mattila et al. | Pain | Patient population |
| Tomkins-Lane et al. | Journal of back and musculoskeletal rehabilitation | Not physical functioning prognostic factors |
| Hägg et al. | European Spine Journal | Not physical functioning prognostic factors |
| Hwang et al. | Musculoskeletal science & practice | Study design |
| Doering et al. | Urogynecology | Patient population |
| Cecchi et al. | European Journal of Physical and Rehabilitation Medicine | Not physical functioning prognostic factors |
| Storheim et al. | Journal of Rehabilitation Medicine | Study design |
| Ash et al. | Bulletin of the American Academy of Psychiatry and the Law | Not physical functioning prognostic factors |
| Hiebert et al. | Spine Journal | Not physical functioning prognostic factors |
| Haukka et al. | Scandinavian Journal of Work, Environment and Health | Not physical functioning prognostic factors |
| Lindell et al. | BMC Family Practice | Study design |
| Sariyildiz et al. | Journal of back and musculoskeletal rehabilitation | Not physical functioning prognostic factors |
| Mesbah et al. | Brain: a journal of neurology | Aim of study is not relevant |
| Sánchez-Mariscal et al. | Spine Journal | Study design |
| Wada et al. | BMC geriatrics | Not physical functioning prognostic factors |
| Sun et al. | Zhonghua lao dong wei sheng zhi ye bing za zhi | Patient population |
| Steffens et al. | European Spine Journal | Not physical functioning prognostic factors |
| Kohlboeck et al. | The Clinical journal of pain | Not physical functioning prognostic factors |
| Konstantinou et al. | The Spine Journal | Not physical functioning prognostic factors |
| Crawford et al. | Spine Journal | Not physical functioning prognostic factors |
| Holtermann et al. | Scandinavian Journal of Work, Environment and Health | Not physical functioning prognostic factors |
| Bekkering et al. | Spine | Not physical functioning prognostic factors |
| Paulsen et al. | Neurosurgery | Not physical functioning prognostic factors |
| Sigurdsson et al. | Acta Orthopaedica | Not physical functioning prognostic factors |
| van den Heuvel et al. | Scandinavian Journal of Work, Environment and Health | Not physical functioning prognostic factors |
| Cruz et al. | PLoS ONE | Not physical functioning prognostic factors |
| Kluszczynski et al. | Polish Annals of Medicine | Aim of study is not relevant |
| Borge et al. | Journal of Manipulative and Physiological Therapeutics | Study design |
| Pedersen et al. | North American Spine Society Journal | Not physical functioning prognostic factors |
| Coric et al. | Journal of Neurosurgery: Spine | Study design |
| Kagaya et al. | Journal of Orthopaedic Science | Not physical functioning prognostic factors |
| Ferguson | ProQuest Dissertations and Theses | Not physical functioning prognostic factors |
| Marras et al. | Spine | Aim of study is not relevant |
| Gracovetsky | Journal of Bodywork and Movement Therapies | Aim of study is not relevant |
| Oliveri et al. | Spine | Study design |
| Ardeshiri | Neuromodulation | Aim of study is not relevant |
| Williams et al. | Journal of Clinical Epidemiology | Study design |
| Pocovi et al. | Musculoskeletal Science and Practice | Not physical functioning prognostic factors |
| Oesch et al. | Physikalische Medizin Rehabilitationsmedizin Kurortmedizin | Not in English language |
| Alsuflany | ProQuest Dissertations and Theses | Study design |
| Yagci et al. | Archives of gerontology and geriatrics | Study design |
| Chen Y.-A. et al. | Physiotherapy (United Kingdom) | Aim of study is not relevant |
| Schiphorst Preuper et al. | European Spine Journal | Study design |
| Ferreira et al. | Manual Therapy | Study design |
| Hira et al. | Scientific Reports | Aim of study is not relevant |
| Elabd et al. | Journal of Bodywork and Movement Therapies | Study design |
| Protas et al. | Spine | Aim of study is not relevant |
| Capodaglio | Europa Medicophysica | Aim of study is not relevant |
| Kumar et al. | European Spine Journal | Aim of study is not relevant |
| Pozzobon et al. | PLoS ONE | Not physical functioning prognostic factors |
| Bäcker et al. | European Spine Journal | Study design |
| Papić et al. | Studies in Health Technology and Informatics | Not physical functioning prognostic factors |
| LeFort et al. | Archives of Physical Medicine and Rehabilitation | Not physical functioning prognostic factors |
| Halonen J.I. et al. | Spine | Not physical functioning prognostic factors |
| Shi et al. | Medicine | Aim of study is not relevant |
| Andersen J.H. et al. | Arthritis and Rheumatism | Aim of study is not relevant |
| Tubach F. et al. | Journal of Occupational and Environmental Medicine | Not physical functioning prognostic factors |
| Wu H. et al. | Journal of Pain Research | Study design |
| Kääriä et al. | European Journal of Pain | Patient population |
| Ingemarsson et al. | Scandinavian Journal of Rehabilitation Medicine | Not physical functioning prognostic factors |
| Master et al. | BMC Musculoskeletal Disorders | Not physical functioning prognostic factors |
| Ueberall et al. | Current medical research and opinion | Aim of study is not relevant |
| Pinto et al. | European Journal of Pain | Not physical functioning prognostic factors |
| Nielsen J. et al. | PeerJ | Aim of study is not relevant |
| Bumann et al. | Spine | Aim of study is not relevant |
| Gautschi et al. | Pain | Aim of study is not relevant |
| Watanabe et al. | Journal of Orthopaedic Science | Aim of study is not relevant |
| Kasis A.G. | Spine | Aim of study is not relevant |
| Eriksen W. | Occupational medicine (Oxford, England) | Patient population |
| Danielsson A.J. | Spine | Patient population |
| Ferguson S.A. | Journal of occupational rehabilitation | Not physical functioning prognostic factors |
| Maldaner et al. | Neurosurgery | Aim of study is not relevant |
| Lubetzky A.V. | The Journal of the American Academy of Orthopaedic Surgeons | Study design |
| Melloh M. | Spine | Full text not found |
| Fujita et al. | Journal of orthopaedic science: official journal of the Japanese Orthopaedic Association | Study design |
| Winkelmuller et al. | Journal of neurological surgery. Part A, Central European neurosurgery | Aim of study is not relevant |
| Dagi et al. | Journal of neurosurgery | Study design |
| Woznowski-Vu et al. | European Journal of Pain (United Kingdom) | Aim of study is not relevant |
| Trolle et al. | Journal of Orthopaedic and Sports Physical Therapy | Not physical functioning prognostic factors |
| Pind R. | Spine | Not physical functioning prognostic factors |
| Balague F. et al. | European Spine Journal | Patient population |
| Frost | Physiotherapy | Study design |
| Lemmers et al. | Musculoskeletal Science and Practice | Study design |
| Flynn et al. | Archives of physical medicine and rehabilitation | Aim of study is not relevant |
| Stubbs et al. | Pain Medicine (United States) | Study design |
| Chang et al. | Arthritis and rheumatism | Patient population |
| Tarnanen et al. | Disability and rehabilitation | Not physical functioning prognostic factors |
| Bentsen H. | Spine | Study design |
| Aghilinejad M. | Medical Journal of the Islamic Republic of Iran | Not physical functioning prognostic factors |
| Nim C.G. | Scientific Reports | Study design |
| Pakkiri Mohamed S.H. | Clinical Rheumatology | Study design |
| Yang S. | Journal of Back and Musculoskeletal Rehabilitation | Study design |
| Gencay-Can A. | European Journal of Physical and Rehabilitation Medicine | Study design |
| Denteneer L. | Journal of Back and Musculoskeletal Rehabilitation | Study design |
| Cegri F. | BMC Geriatrics | Patient population |
| Heymans M.W. | Spine Journal | Not physical functioning prognostic factors |
| Oliveri M. | Spine | Full text not found |
| Gross D.P. | Spine | Study design |
| Gross D.P. | Spine | Study design |
| Stenholm S. | Journals of Gerontology Series A: Biological Sciences & Medical Sciences | Patient population |
| Andrews N.E. | Clinical Journal of Pain | Aim of study is not relevant |
| Vanti C. | Journal of manipulative and physiological therapeutics | Aim of study is not relevant |
| Abdelraouf O. | International journal of sports physical therapy | Aim of study is not relevant |
| Haddas R. | Spine | Patient population |
| Imagama S. | Spine | Patient population |
| Boody B. | Pain medicine (Malden, Mass.) | Not physical functioning prognostic factors |
| Onoyama-Ball M.L. | ProQuest Dissertations and Theses | Study design |
| Acar et al. | Turkish Journal of Physiotherapy and Rehabilitation | Not in English language |
| Fitzgerald et al. | Neurourology and urodynamics | Patient population |
| Glauser et al. | Neurosurgery | Aim of study is not relevant |
| Kim et al. | Spine | Aim of study is not relevant |
| Schenk et al. | European journal of applied physiology | Aim of study is not relevant |
| Cabrera et al. | European spine journal : official publication of the European Spine Society, the European Spinal Deformity Society, and the European Section of the Cervical Spine Research Society | Aim of study is not relevant |
| Yeung TSM et al. | Journal of Orthopaedic & Sports Physical Therapy | Study design |
| Balkic Widmann J. et al. | New Armenian Medical Journal | Study design |
| Atya | Journal of advanced research | Study design |
| Ferguson et al. | Proceedings of the Human Factors and Ergonomics Society | Aim of study is not relevant |
| Mitchell B. | Journal of Science and Medicine in Sport | Aim of study is not relevant |
| Dobson | Journal of physiotherapy | Aim of study is not relevant |
| Tarrant et al. | Spine | Patient population |
| Alexopulos | ProQuest Dissertations and Theses | Study design |
| Coyle et al. | Journal of Geriatric Physical Therapy | Not physical functioning prognostic factors |
| Bachman S. et al. | Physikalische Medizin Rehabilitationsmedizin Kurortmedizin | Full text not found |
| Lopes T.J.A. et al. | Journal of Science and Medicine in Sport | Patient population |
| Balaguier et al. | PloS one | Patient population |
| Hicks et al. | The journals of gerontology. Series A | Not physical functioning prognostic factors |
| Ilves O.E. et al. | Neurospine | Aim of study is not relevant |
| Burdorf A. | Clinical biomechanics (Bristol, Avon) | Patient population |
| Cho K.H. | Annals of rehabilitation medicine | Patient population |
| Lindsay D.M. | North American journal of sports physical therapy : NAJSPT | Aim of study is not relevant |
| Fonseka R. | Journal of spine surgery (Hong Kong) | Study design |
| Shaw J. | Journal of electromyography and kinesiology : official journal of the International Society of Electrophysiological Kinesiology | Aim of study is not relevant |
| Sakaguchi T. | Journal of clinical medicine | Study design |
| Carayannopoulos A.G. | Journal of Pain Research | Aim of study is not relevant |
| Dubick M.N. | Journal of Pain Research | Aim of study is not relevant |
| Teixeira da Cunha-Filho I. | Physiotherapy theory and practice | Aim of study is not relevant |
| Komodikis G. | Clinical Neurosurgery | Patient population |
| Snyder | ProQuest Dissertations and Theses | Aim of study is not relevant |
| Sung | ProQuest Dissertations and Theses | Not physical functioning prognostic factors |
| Perret et al. | Archives of physical medicine and rehabilitation | Aim of study is not relevant |
| Weisman | Technology and Disability | Aim of study is not relevant |
| Lu et al. | Chinese Journal of Tissue Engineering Research | Aim of study is not relevant |
| Grelat et al. | World neurosurgery | Aim of study is not relevant |
| Tang et al. | Seminars in Spine Surgery | Not physical functioning prognostic factors |
| Hayden et al. | Best Practice and Research: Clinical Rheumatology | Not physical functioning prognostic factors |
| Teyhen et al. | Clinical Orthopaedics and Related Research | Patient population |
| Cook et al. | Physical therapy | Not physical functioning prognostic factors |
| Pearson et al. | Spine | Not physical functioning prognostic factors |
| Euro et al. | Scientific reports | Not physical functioning prognostic factors |
| Spittler et al. | Clinical Journal of Sport Medicine | Full text not found |
| Andrade et al. | Journal of the American Geriatrics Society | Study design |
| Cheng et al. | Journal of occupational and environmental medicine | Study design |
