# Supplementary material for: Physical measures of physical functioning as prognostic factors to predict outcomes in low back pain: A systematic review and narrative synthesis
Source: PLoS One. 2025 Oct 28;20(10):e0335535. doi: 10.1371/journal.pone.0335535 (PMC12561921; doi:10.1371/journal.pone.0335535)
Supplement: S7 File — (DOCX) [file pone.0335535.s007.docx]

**Potential predictors in Individual studies**

**Higher Value of these test predict good outcomes**

1. Sit up test
2. Thoracolumbar rotation
3. TUG
4. Quadricep strength
5. Average hip rotation equal or greater than 25 (bilateral)
6. Trunk flexion performance test
7. Trunk extension performance test
8. 6 Min walk test
9. Maximal side bending
10. FCE performance test
11. Lifting ability test
12. Villager test
13. Oesch test
14. 10 m self paced walking with smart shoes
15. Total walking time
16. Hip internal rotation
17. Step test
18. Physical activity level
19. Moderate to vigorous PA (Time)
20. Daily Step count
21. Hip abductor muscle endurance
22. FTSTs (5 times sit to stand test)

**Lower Value of this test predicts good outcomes**

1. Sedentary PA (Time)
